# Adverse childhood experiences and risk of non-mental medical diseases in adulthood: an umbrella review

**DOI:** 10.1016/j.eclinm.2026.103987

**Published:** 2026-05-22

**Authors:** Alicia Valiente-Gómez, Joana Bücker, Marta Fontana-McNally, Daniela L. Gatto, Daniel Guinart, Ana Moreno-Alcázar, Carlos Campos Rodríguez, Cristina de Córdoba Gil, Frank Padberg, Helena Pardina Torner, Joaquim Radua, Victor Pérez-Solà, Paolo Fusar-Poli, Enric Vilajosana, Bridget Hogg, Adriane R. Rosa, Benedikt L. Amann

**Affiliations:** a Hospital del Mar Research Institute, Barcelona, Spain; b Mental Health Institute, Hospital del Mar, Barcelona, Spain; c Centro de Investigación Biomédica en Red de Salud Mental (CIBERSAM), Instituto Carlos III, Madrid, Spain; d Programa de Pós-Graduação em Ciências Médicas – PPGCM, Universidade do Vale do Taquari – UNIVATES, Brazil; e Faculty of Medicine and Life Sciences, Universitat Pompeu Fabra, Barcelona, Spain; f Department of Psychiatry, The Donald and Barbara Zucker School of Medicine at Northwell/Hofstra University, New York, USA; g Department of Psychiatry and Psychotherapy, LMU University Hospital, Munich, Germany; h DZPG (German Center for Mental Health), Partner Site Munich-Augsburg, Munich, Germany; i Post-doctoral Programme, Campus Bellvitge, Universitat de Barcelona, Barcelona, Spain; j Institut d’Investigacions Biomèdiques August Pi i Sunyer (IDIBAPS), Barcelona, Spain; k Department of Clinical Neuroscience, Karolinska Institutet (KI), Sweden; l Department of Psychosis Studies, Institute of Psychiatry, Psychology and Neuroscience, King's College London, United Kingdom; m Department of Brain and Behavioural Sciences, University of Pavia, Italy; n Outreach and Support in South London (OASIS) Service, South London and Maudsley (SLaM) NHS Foundation Trust, UK; o Laboratory of Molecular Psychiatry, Hospital de Clínicas de Porto Alegre (HCPA), Porto Alegre, RS, Brazil; p Departamento de Farmacologia, Instituto de Ciéncias Básicas de Saúde, Universidade Federal do Rio Grande do Sul, Brazil; q Postgraduate Program in Psychiatry and Behavioral Sciences, Federal University of Rio Grande do Sul (UFRGS), Porto Alegre, RS, Brazil

**Keywords:** Childhood trauma, Medical disease, Cardiovascular disease, Diabetes, Cancer

## Abstract

**Background:**

This umbrella review evaluates adverse childhood experiences (ACEs) as etiological risk factors for non-mental medical diseases.

**Methods:**

We systematically searched PubMed, EMBASE and WoS up to 30 April 2025, following PRISMA, PRIOR and MOOSE guidelines. Eligible studies were meta-analyses or systematic reviews investigating associations between ACEs and non-mental medical disease, with control groups and adequate data for extraction. Studies on congenital disorders or without relevant data were excluded. Methodological quality was assessed using AMSTAR. The protocol was registered in PROSPERO (CRD42022384104). Meta-analytic data were synthesised with random-effects models to calculate odds ratios (OR) with confidence intervals and p-values. Heterogeneity was assessed with I^2^, small-study effects with the Egger test, and 95% prediction intervals were calculated. Evidence credibility was graded according to Ioannidis’ criteria.

**Findings:**

We included 36 reviews/meta-analyses covering 250 non-duplicated studies with 6,064,006 participants (412,760 cases and 5,651,246 controls). There was highly suggestive evidence (Class II) linking any ACE to any non-mental medical disease (OR = 1.57; 95% CI: 1.49, 1.66). Strong associations were found between any non-mental medical disease and abuse of any type (OR = 1.61), physical abuse (OR = 1.59), sexual abuse (OR = 1.58) and bullying (OR = 2.04). Regarding specific diseases, highly suggestive associations were identified for headache (OR = 1.91), irritable bowel syndrome (OR = 1.79), diabetes (OR = 1.67), and cardiovascular disease (OR = 1.46). Convincing evidence emerged for a modest association between ACEs and cardiovascular disease (OR = 1.19) and endocrine/metabolic disorders (OR = 1.62) in prospective studies.

**Interpretation:**

ACEs are significant risk factors for adult non-mental medical diseases, underscoring the importance of early intervention and prevention for vulnerable groups. Early stressors have lasting physical health impacts. Limitations include reliance on self-reported trauma and heterogeneous study designs; prospective data showed lower bias, reinforcing the need for rigorous research. Future studies should explore genetic, environmental, and resilience factors that may moderate medical risks among individuals exposed to ACEs.

**Funding:**

No funding was received.

**Research in context**

**Evidence before this study**

Exposure to adverse experiences during childhood is common, affecting an estimated 60% of the adult population, and constitutes a powerful transdiagnostic predictor of psychopathology. Although strong evidence links adverse childhood experiences (ACEs) to mental disorders and mental disorders to non-mental medical diseases, a comprehensive synthesis directly addressing the relationship between ACEs and physical health outcomes is still lacking. We searched EMBASE, Web of Science, and PubMed from database inception to 19/01/2023, and updated the search to 30/04/2025, for studies published in any language, using search terms developed in collaboration with a specialist librarian. Previous meta-analyses and systematic reviews have shown a direct association between ACEs and physical illness. However, we did not identify any existing umbrella review of systematic reviews and meta-analyses on ACEs and medical conditions.

**Added value of this study**

Drawing on data from over 6 million participants, this umbrella review examined the association between ACEs and 15 medical outcomes. We found highly suggestive (Class II) evidence that ACEs are broadly associated with non-mental medical diseases. The strongest associations were observed for overall medical morbidity linked to physical or sexual abuse, or bullying. Specific conditions with highly suggestive evidence included headache, irritable bowel syndrome, diabetes, and cardiovascular disease. Convincing evidence supported ACEs as risk factors for cardiovascular disease, while prospective studies also implicated ACEs in endocrine and metabolic disorders.

**Implications of all the available evidence**

The evidence that ACEs are linked to a wide range of non-mental medical diseases underscores the value of early prevention and intervention for populations at risk. Addressing the long-term health consequences of childhood trauma through preventive strategies is crucial to improve population health and reduce pressure on healthcare systems. These findings point to the urgent need for targeted programs and policies that strengthen collaboration between primary care and mental health services, supported by adequate funding and a comprehensive, cross-sectoral approach.

## Introduction

Multiple factors have been associated with the pathogenesis of non-mental medical diseases such as cancer, cardiovascular, gastrointestinal, urogynaecological, neurological or autoimmune diseases. Growing evidence highlights the role of psychosocial variables, such as social support, coping styles, and socioeconomic status as risk factors for their onset,^1^^,^^2^ and poor mental health can exacerbate physical symptoms.^3^ Some non-mental medical diseases in adulthood may originate in early-life physiological processes,^4^ with cardiovascular diseases (CVD)—a major global health burden—being associated with trauma exposure in childhood.^5^

Adverse childhood experiences (ACEs), including physical, sexual or emotional abuse, neglect, parental separation, or loss, affect about 60% of adults^6^ and represent the strongest transdiagnostic risk factors for mental disorders.^7^^,^^8^ ACEs may produce chronic stress dysregulation,^9^ whereby cumulative or extreme stress during sensitive developmental stages can sensitise the neuroendocrine system, leading to prolonged activation of the Hypothalamic-Pituitary-Adrenal (HPA) axis and autonomic nervous system.^9^ Acute stress can transiently enhance immune responses and promote protection during infection; in contrast, chronic stress leads to dysregulation or suppression of immune functions.^10^ Such alterations, particularly in early childhood and adolescence,^11^ affect cortisol regulation and increase vulnerability to both mental and non-mental medical diseases.^9^ Evidence shows ACEs nearly triple the risk of any mental disorder,^8^ while also being associated with medical disease. Posttraumatic stress disorder (PTSD), the prototypical trauma-related disorder, has been linked to elevated risk of cardiovascular events^12^ and autoimmune conditions, including irritable bowel syndrome, rheumatoid arthritis, and multiple sclerosis.^13^ Immunological changes in PTSD, such as increased leucocyte and T-cell counts, may underlie this association.^14^^,^^15^

While robust evidence supports links between ACEs and mental disorders,^7^^,^^8^ and between mental disorders and medical disease,^3^ no systematic synthesis has yet examined the direct association between ACEs and non-mental medical diseases. This paper therefore aimed to conduct an umbrella review of systematic reviews and meta-analyses on ACEs and non-mental medical diseases, assessing the strength of overall and disorder-specific associations, as well as the influence of sex, ACE subtypes, and methodological biases (e.g., retrospective vs. prospective studies).

## Methods

### Search strategy and selection criteria

This umbrella review^16^ analysed data from published meta-analyses and extracted findings from systematic reviews to examine the association between ACE and non-mental medical diseases. It followed PRISMA,^17^ PRIOR^18^ and MOOSE^19^ guidelines and was prospectively registered in PROSPERO (CRD 42022384104) (https://www.crd.york.ac.uk/PROSPERO/view/CRD42022384104). No amendments to the protocol have been made at any point of the study. Searches were conducted in EMBASE, Web of Science, and PubMed from database inception to 19/01/2023, and updated to 30/04/2025. Search terms, developed with a specialist librarian from the Universidade Federal do Rio Grande do Sul, Brazil, are detailed in Supplementary Information 1.

Inclusion criteria were: (1) systematic review and/or meta-analysis; (2) evaluation of ACE in relation to at least one non-mental medical disease; (3) inclusion of a control group (i.e. individuals without the non-mental medical disease); (4) publication in any language with English abstract; (5) sufficient data for analysis. Exclusion criteria were: (1) reviews not reporting associations between ACE and non-mental medical disorders; (2) insufficient relevant data; (3) focus on congenital disorders. Non-mental medical diseases were defined as conditions defined by standardised diagnostic criteria (e.g., ICD-11) or validated clinical definitions to ensure consistency. Isolated symptoms that did not constitute a formal diagnosis were excluded. Screening was conducted in Rayyan in two stages (title/abstract, full text) by two independent reviewers, with disagreements resolved by consensus with a third researcher. A list of excluded studies is provided in Supplementary Information 2. Review quality was assessed with the AMSTAR tool, applied independently by two reviewers, with discrepancies resolved through consensus with a third.

For each eligible review or meta-analysis, data were extracted from the included primary studies. When information was incomplete, original articles were consulted. Data was extracted for each primary study with relevant data included in the reviews and meta-analyses. We then performed our own analysis using the data from each primary study. Factors (ACE and non-mental medical diseases) were extracted as defined in the corresponding meta-analysis or systematic review.

Duplicated studies were screened for by a review of all primary studies by lead author and year. When there was a possible duplicate (i.e. the same lead author and year), the authors reviewed the manuscript to check if this was the same article. In that case, we checked if the same population has been duplicated or not. One meta-analysis may have included e.g., the same population but with a focus on cancer, while another focused on cardiovascular disease, and therefore the extracted data were not considered as duplicated. In case of duplicate, this article was removed. This process was carried out by 2 independent researchers (BH and DG) in a blind screening process and then any discrepancies were resolved in a consensus meeting.

Extracted variables included: first author, year of publication, type and measure of ACE (prospective/retrospective), type and measure of medical disorder (prospective/retrospective), statistical measure (e.g., Odds Ratio) with confidence interval, number of cases (individuals with a non-mental medical disorder) and controls (individuals without the non-mental medical disorder), number of exposed and non-exposed individuals, and means with standard deviations. Data extraction was performed independently and blindly by two researchers, with discrepancies resolved through consensus with a third. When data remained insufficient, study authors were contacted; if no information was obtained, the study was excluded from the final analyses.

The dataset generated and analysed during the current study, including data extraction tables and supplementary analyses, is available in the Zenodo repository (https://doi.org/10.5281/zenodo.16921572).

### Data analysis

The primary objective was to examine the overall association between any ACE and any non-mental medical diseases. Secondary objectives included assessing links between specific ACE and any non-mental medical diseases, as well as any ACE and specific non-mental medical diseases.

A random-effects meta-analysis was performed using measures of association (e.g., Odds Ratio [OR], Risk Ratio [RR]). Each meta-analytic unit was defined as a unique combination of an ACE exposure and a non-mental medical disease outcome (hereafter referred to as a factor; e.g., emotional abuse–cardiovascular disease). For each factor, all relative measures of association were converted to OR prior to pooling to ensure comparability across studies. The confidence interval and p-value were calculated, along with I^2^ for heterogeneity, Egger's test for small-study effects, 95% prediction intervals, and a binomial test comparing observed vs. expected significant results.

These data were used to estimate the strength of associations and credibility of the evidence according to Ioannidis’ criteria.^20^ We used the Ioannidis criteria to grade the evidence, considering effect size, heterogeneity, publication bias, and prediction intervals. Evidence can be classified as convincing (class I), highly suggestive (class II), suggestive (class III), or weak (class IV). Only results graded as convincing or highly suggestive are presented in the main article; others appear in the supplementary material (see Table 1 for criteria).

**Table 1:** Ioannidis’ classification of evidence.

Evidence level	Class	Description
Convincing	I	Large number of cases (n > 1000) Highly statistically significant association (p < 10–6) Low between-study heterogeneity I^2^ < 50% 95% prediction interval excluding null hypothesis Absence of small-study effects (Eggers' test p > 0.05) Absence of excess significance bias (binomial test p > 0.05)
Highly suggestive	II	Large number of cases (n > 1000) Highly statistically significant association (p < 10–6) Largest study has a statistically significant effect
Suggestive	III	Large number of cases (n > 1000) Statistically significant association (p < 0.001)
Weak	IV	No specific number of cases Statistically significant association (p < 0.05)

For the primary objective, a single pooled analysis was conducted combining all ACE types and non-mental medical diseases to estimate the overall association. For the secondary objective, factors were obtained by individual associations between ACE and non-mental medical diseases (e.g., emotional abuse with diabetes) when at least three studies addressed the association; additionally ACE were grouped into broader categories–abuse (physical, emotional, sexual), neglect (physical, emotional), and household dysfunction (including family mental illness, caregiver loss, separation, imprisonment, substance use, financial difficulties, or family dysfunction)–and non-mental medical diseases grouped according to the International Classification of Diseases 11.^17^

We performed a subgroup analysis including only studies with prospective measures, as these provide stronger evidence^18^ and reduce risk of recall bias in reporting.^19^ Separate subanalyses were conducted for studies measuring both trauma and disease prospectively, and for those with prospective disease outcomes but retrospectively assessed ACEs. Additional subgroup analyses examined female-only and male-only samples to explore sex effects.

### Role of funding source

No funding was received.

## Results

**Fig. 1:**
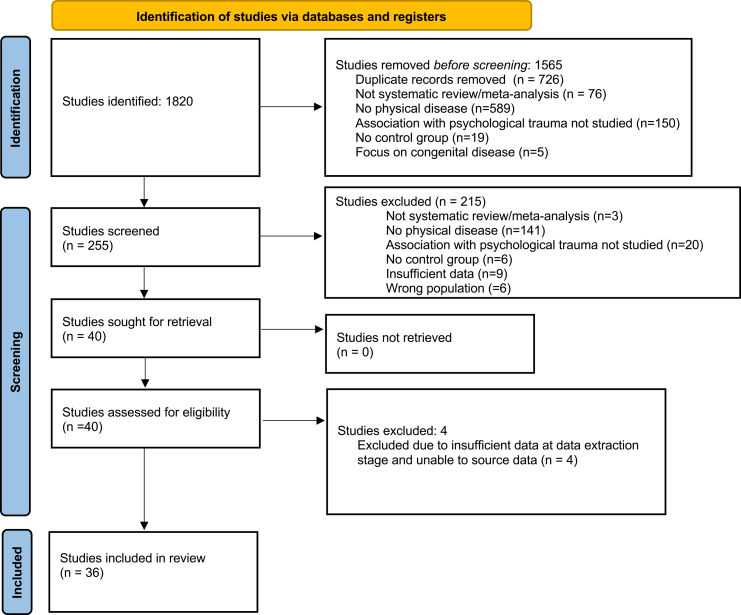
**PRISMA 2020 Flow Diagram for new systematic reviews which included searches of databases and registers only**. Source: Page MJ et al. BMJ 2021; 372:n71. https://doi.org/10.1136/bmj.n71. This work is licenced under CC BY 4.0. To view a copy of this licence, visit https://creativecommons.org/licenses/by/4.0/.

The initial search until 19/01/2023 returned 1293 results, which were reduced to 751 once duplicates were removed. Of these, following title/abstract review, 119 articles passed to the second stage for full text review, of which 26 met the inclusion criteria.^21^, ^22^, ^23^, ^24^, ^25^, ^26^, ^27^, ^28^, ^29^, ^30^, ^31^, ^32^, ^33^, ^34^, ^35^, ^36^, ^37^, ^38^, ^39^, ^40^, ^41^, ^42^, ^43^, ^44^, ^45^, ^46^ The search was then updated to include articles until 30th April 2025. This search returned 527 results, which were reduced to 343 once duplicated were removed. Of these, 136 passed the initial stage of screening, of which 10 met the inclusion criteria,^47^, ^48^, ^49^, ^50^, ^51^, ^52^, ^53^, ^54^, ^55^, ^56^ meaning a final total of 36 included meta-analyses and systematic reviews. A PRISMA flow diagram can be seen in Fig. 1.

These articles included 250 individual studies which met our inclusion criteria, with a total of 6,046,006 participants (412,760 cases and 5,651,246 controls). This included 15 types of non-mental medical diseases (cancer, cardiovascular, diabetes, dysmenorrhoea, female genital disease, headache, human immunodeficiency virus (HIV), irritable bowel syndrome (IBS), multiple sclerosis, obesity, pelvic and genital pain, respiratory, urinary tract infection, somatic pain and other), which were grouped into 11 types of non-mental medical diseases according to ICD-11 categories: circulatory system, respiratory system, neoplasms, general symptoms of pain, digestive system, infectious, urinary system, genitourinary system, endocrine/nutritional/metabolic, and other diseases. Pelvic and genital pain as well as somatic pain as been included as categories as both are coded in the ICD-11 and are clinically highly relevant. The disease names reported in the original reviews were mapped to ICD-11 categories by two independent researchers. Any discrepancies in the coding were discussed and resolved by consensus with a third researcher. The categories of ACE represented in the included studies were: abuse (comprising measures of emotional abuse, physical abuse, sexual abuse, or a combination of them); neglect (comprising emotional neglect and physical neglect, either separately or together); and family/household stressors (comprising divorce/separation/death of a parent, having an incarcerated parent, family dysfunction, financial difficulties, parental unemployment, witnessing intimate partner violence in the home, witnessing any form of violence in the home, or having a family member with mental illness or substance use problems in the home), bullying (referring to victimisation in a school setting and/or peer-to-peer), and finally, there was a general category for non-specified ACE. The dataset is publicly available.

Regarding trauma measures, 47 of the 459 associations between an ACE and a non-mental medical disease studied included non-self-report measures, such as record-based information on trauma (child protection services reports, medical reports, or data from the civil register) or reports by teachers, parents, or medical professionals. This information can be seen in Table 2, along with a description of each of the included studies and the quality score according to the AMSTAR.

**Table 2:** Overview of included studies.

First author, year	Type of article	Type of trauma	K (total)	K (prospective studies—trauma and disease)	K3 (prospective studies—disease)	Self-reported trauma measures only	Inclusion of objective measures^a^	Diagnosis	AMSTAR score
Afari 2014	MA	Abuse	3	0	0	3	0	Somatic pain	8
Amiri 2024	MA	ACE	23	2	4	16	7	Obesity	6
Berndt 2024	SR	Abuse	14	6	7	14	0	Female genital disease	7
		ACE	2	0	0	2	0		
		Neglect	3	0	0	3	0		
		Household Dysfunction^b^	6	0	0	6	0		
Chen 2023	MA	Abuse	20	0	3	12	8	Cardiovascular	8
Danese 2014	MA	Abuse	3	0	0	2	1	Obesity	8
		ACE	2	0	0	1	1		
		Neglect	1	0	1	0	1		
Devuyst 2021	SR	ACE	3	0	0	3	0	Diabetes	4
		Household Dysfunction	1	0	0	1	0		
Duan 2021	MA	Abuse	7	0	0	7	0	Respiratory disease	8
		ACE	3	0	1	3	0		
		Witness violence in the home	3	0	0	3	0		
Elsenburg 2015	SR	ACE	8	0	3	8	0	Obesity	9
Gini 2014	MA	Bullying	9	1	1	9	0	Headache	9
Hassam 2020	MA	Sexual abuse	7	0	0	7	0	Pelvic/genital pain	7
Hauser 2011	MA	Abuse	5	0	0	5	0	Somatic pain	7
Hemmingsson 2014	MA	Abuse	35	0	3	35	0	Obesity	5
		Neglect	3	0	0	3	0		
Holman 2016	SR	ACE	6	0	2	4	2	Cancer	4
		Abuse	13	0	0	13	0		
		Household Dysfunction	1	0	0	1	0		
Hu 2021	MA	Abuse	4	0	0	4	0	Cancer	7
		ACE	5	0	3	5	0		
		Household Dysfunction	8	0	0	8	0		
		Witness violence in the home	3	0	0	3	0		
Huang 2015	MA	Abuse	4	2	0	4	0	Diabetes	7
		ACE	3	1	1	2	1		
		Neglect	1	0	0	1	0		
		Other	1	1	1	1	0		
Jacquet-Smailovic 2022	MA	ACE	9	0	1	9	0	Cardiovascular	10
Jakubowski 2021	MA	Abuse	9	0	1	8	1	Cardiovascular	7
								Diabetes	
Jianyi Liu 2024	MA	Abuse	17	0	1	17	0	Headache	10
Junaid 2023	SR	Abuse	7	0	0	7	0	Irritable bowel syndrome	8
Joshee 2022	MA	ACE	15	0	2	14	1	Irritable bowel syndrome	8
Kaleycheva 2021	MA	Abuse	15	0	0	15	0	Diabetes	10
		ACE	3	0	0	3	0		
Lenover Moya 2025	MA	Abuse	15	2	2	15	0	Irritable bowel syndrome	7
		ACE	3	0	0	3	0		
Lloyd 2012	MA	Abuse	4	0	1	4	0	HIV	7
Lopes 2020	MA	ACE	14	0	3	13	1	Cancer	6
								Respiratory	
Moussaoui 2022	SR	Abuse	5	0	0	5	0	Dysmenorrhea/Pelvic/genital pain	5
		ACE	2	0	0	2	0		
		Bullying	1	0	0	1	0		
		Other	1	0	0	1	0		
Mikio Moriya 2022	SR	ACE	7	3	3	5	2	Obesity	8
		Household Dysfunction	1	1	1	1	0		
Oh 2018	SR	Abuse	5	5	5	0	5	Obesity	10
		Household Dysfunction	7	3	3	4	3	Respiratory disease	
		Other	1	0	0	0	1		
Paras 2009	MA	Abuse	3	0	1	3	0	Pelvic/genital pain	5
								Obesity	
								Other	
Polick 2022	SR	Abuse	5	0	2	2	0	Multiple Sclerosis	5
		Household Dysfunction	2	0	0	0	2		
		Neglect	1	0	0	1	0		
Provencher 2019	MA	Household Dysfunction	5	0	1	4	1	Cardiovascular	6
								Genitourinary disease	
								Obesity	
								Other	
								Respiratory disease	
Rehan 2023	SR	ACE	4	3	1	4	0	Multiple Sclerosis	7
		Abuse	4	3	0	4	0		
Schroeder 2021	SR	Abuse	6	0	0	4	2	Obesity	6
		Household Dysfunction	2	1	1	1	1		
		Witness violence in the home	3	1	3	0	3		
Selai 2023	SR	Abuse	3	0	0	3	0	Genitourinary disease	7
Souama 2023	MA	ACE	13	0	10	13	0	Cardiovascular	6
Zhang 2022	MA	Abuse	10	0	2	10	0	Diabetes	9
		Bullying	1	0	0	1	0		
		Household Dysfunction	13	0	6	13	0		
		Neglect	3	0	0	3	0		
Zhou 2024	MA	ACE	12	0	2	11	1	Obesity	8
		Abuse	14	0	10	13	1		
		Household dysfunction	2	0	0	2	0		
		Neglect	2	0	2	1	1		

Abbreviations: HIV, Human Immunodeficiency Virus; K, number of studies; MA, meta-analysis; SR, systematic review.

a Objective measures included were: Child Protection Services Records, Medical Records, Civil Records, Parent Report, Teacher Report.

b Household Dysfunction comprises: “family_mental_illness”; “divorce/separation/death”; “family_dysfunction”; “family_separation”; “financial_difficulties”; “incarcerated_family_member”; “substance_use_family”; “household_dysfunction”.

### Overall association between any type of ACE and any type of non-mental medical disease

Combining all types of ACE, and all types of non-mental medical disease, the analysis showed highly suggestive (Class II) evidence of an association between all types of ACE and any non-mental medical disease (OR = 1.57; 95% CI: 1.49, 1.66); please see Supplementary Figure S1 and Supplementary Table S1).

### Association between specific types of ACE and any type of non-mental medical disease

**Fig. 2:**
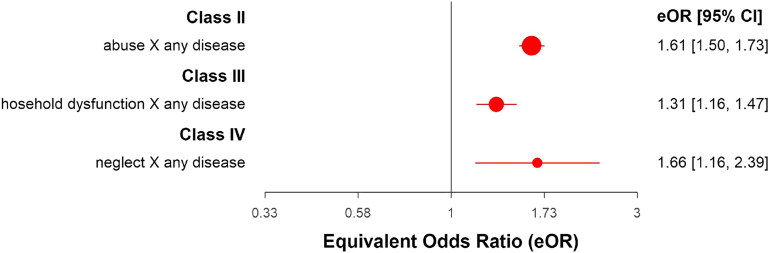
**Class II, III and IVassociations between specific types of ACE with any physical disease.** ∗For visual purposes, only factors with >2 individual studies are represented in the figure.

**Fig. 3:**
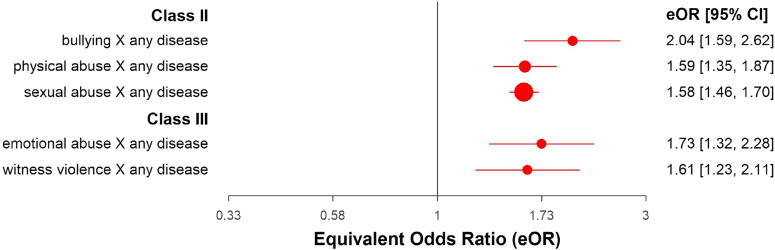
**Class II and Class IIIassociations between different categories of ACE with any physical disease.** ∗For visual purposes, only factors with >2 individual studies are represented in the figure.

Analysis of the association between different types of ACE revealed Class II evidence for abuse of any type (OR = 1.61; 95% CI: 1.47, 1.77), due to risk of bias and the largest study returning a non-significant result (please see Fig. 2 and Supplementary Table S2). There was highly suggestive evidence (Class II) of an association between physical abuse (OR = 1.59; 95% CI: 1.35, 1.87); sexual abuse (OR = 1.58; 95% CI: 1.46, 1.70), and bullying (OR = 2.04; 95% CI: 1.59, 2.62) in relation to any non-mental medical disease (please see Fig. 3, and Supplementary Table S2). These associations did not reach class I evidence because of the presence of heterogeneity, a 95% prediction interval including the null, and potential publication and excess significance biases, except for bullying, which did not reach significance for potential publication biases. Regarding the remaining ACE types (also in Fig. 3), only emotional abuse (OR = 1.73; 95% CI: 1.32, 2.28) and witnessing violence (OR = 1.61; 95% CI: 1.23, 2.11) reached suggestive evidence (Class III). Non-significant or associations of a weak level of evidence can be seen in Supplementary Table S2.

### Association between any type of ACE and specific types of non-mental medical disease

Regarding associations between any type of ACE and specific non-mental medical diseases, there was highly suggestive evidence (Class II) for an association between any type of ACE and headache (OR = 1.91; 95% CI: 1.60, 2.28), IBS (OR = 1.79; 95% CI: 1.45; 2.22), diabetes (OR = 1.67; 95% CI: 1.41, 1.97) and CVD (OR = 1.46; 95% CI: 1.27, 1.66). Other specific diseases showed suggestive or weak evidence only, except for respiratory disease, which showed no significant association (see Supplementary Figure S2, and Supplementary Table S3). After grouping the non-mental medical diseases according to ICD-11 classifications, we found highly suggestive (Class II) evidence for an association between ACE and disorders of the nervous system (OR = 1.84; 95% CI: 1.56, 2.18), digestive system (OR = 1.80; 95% CI: 1.45, 2.22); endocrine/nutritional/metabolic (OR = 1.51; 95% CI: 1.40, 1.64), and circulatory system (OR = 1.46; 95% CI: 1.27, 1.66). These associations, along with other associations with suggestive or weak evidence, or where no significant association was found, can be seen in Supplementary Table S4.

### Association between specific types of ACE and specific types of non-mental medical disease

Regarding associations between specific types of ACE, and specific types of non-mental medical disease, there was highly suggestive evidence (Class II) of an association between headaches and bullying (OR = 2.04, 95% CI: 1.55; 2.69), an association between obesity and sexual abuse (OR = 1.45; 95% CI: 1.28, 1.64) and between obesity and the divorce, separation or death of a caregiver (OR = 1.58; 95% CI: 1.36, 1.83). These associations, as well as evidence for weaker or non-significant associations are presented in Supplementary Table S5 and Supplementary Figure S3. In Supplementary Table S6 the results can be seen once the diseases are grouped into the relevant ICD-11 category.

A table summarising all the Class I and Class II associations between any type of ACE and any type of non-mental medical disorder can be seen in Table 3. The specific studies which were included in the statistical analysis of each association can be seen in Supplementary Tables S7 to S14 (eight tables). Funnel plots were generated for the main analyses showing Class II evidence or above (Supplementary Figures S4–S19).

**Table 3:** Overview of all associations between any type of Adverse Childhood Experience and any type of specific disease meeting Class I and Class II evidence criteria.

ACE type	Disease type	N cases	Class	OR	CI	p-value
Any ACE	Any disease	412,760	II	1.58	[1.43, 1.747]	3.12e-19
Any abuse	Any disease	289,69 7	II	1.60 9	[1.498, 1.728]	4.42e-39
Bullying	Any disease	8487	II	2.03 7	[1.587, 2.616]	2.36e-08
Physical abuse	Any disease	225,47 9	II	1.58 6	[1.346, 1.869]	3.77e-08
Sexual abuse	Any disease	215,99 7	II	1.57 8	[1.464, 1.701]	1.43e-32
Any ACE	Headache	15,898	II	1.90 9	[1.602, 2.276]	5.45e-13
Any ACE	Irritable Bowel Syndrome	11,517	II	1.79 5	[1.449, 2.224]	8.79e-08
Any ACE	Diabetes	36,350	II	1.66 7	[1.411, 1.968]	1.73e-09
Any ACE	Cardiovascular Disease	99,305	II	1.45 5	[1.272, 1.664]	4.43e-08
Bullying	Headache	7923	II	2.04 4	[1.554, 2.688]	3.17e-07
Divorce/separation/death	Obesity	42,577	II	1.57 6	[1.356, 1.833]	3.33e-09
Emotional abuse	Headache	2094	II	1.57 3	[1.358, 1.823]	1.64e-09
Sexual abuse	Obesity	13,550	II	1.44 9	[1.281, 1.639]	3.47e-09

Key. N, number of participants; OR, Odds Ratio; CI, Confidence Interval; ACE, Adverse Childhood Experience.

### Analysis by sex

We carried out further analyses to determine if: 1) there were sex differences in the association between ACE and non-mental medical diseases, and 2) there was a difference in results when the analysis was restricted to only prospective studies providing a higher quality of evidence. The analyses were only considered on factors reaching at least highly suggestive (Class II) evidence to minimise false positive results.

### Sex differences

Analysing the association between any type of ACE and any type of non-mental medical diseases, there was highly suggestive evidence (Class II) of an association (OR = 1.58, 95% CI: 1.43, 1.74) in studies with female-only samples, while in studies with male-only samples there was suggestive evidence (Class III) of an association (OR = 1.42, 95% CI: 1.18, 1.70). Regarding any type of ACE and specific disease, the only significant association with highly suggestive evidence (Class II) was an association between any type of ACE and CVD in males (OR = 1.26; 95% CI: 1.18, 1.34). There was also highly suggestive evidence (Class II) in females for an association between sexual abuse (OR = 1.52; 95% CI: 1.37, 1.69), physical abuse (OR = 1.31; 95% CI: 1.18, 1.45), and any disease. In male-only samples, there was a significant association between sexual abuse and any disease (OR = 1.54; 95% CI: 1.21, 1.97), and physical abuse and any disease (OR = 1.25, 95% CI: 1.09, 1.44), but these were lower quality evidence: suggestive and weak evidence respectively. All results for female-only samples can be seen in Supplementary Table S15, while all results for male-only samples can be seen in Supplementary Table S16.

All analyses considering sex exhibit the same trend in evidence class, both meeting similar OR. However, the analyses of male sex as variable generally met requirements for one or two classes lower evidence than those for female sex. This discrepancy is likely due to smaller sample sizes in the male analyses compared to the female analyses which limits the ability to achieve higher evidence class levels.

### Prospective vs retrospective study design

When the analysis was restricted to studies using a prospective design, there was still highly suggestive evidence for an association with any type of ACE and any type of non-mental medical disease (OR = 1.47, 95% CI: 1.29, 1.68; see Supplementary Table S17). Furthermore, there were some differences in terms of evidence class for some individual results. There was convincing evidence (Class I) of a small but robust association for any type of ACE with circulatory system disease (OR = 1.19; 95% CI: 1.12, 1.26) when disease was measured using a prospective design. Meanwhile, any type of ACE showed convincing evidence (Class I) of an association with endocrine/nutritional/metabolic disease (OR = 1.62; 95% CI: 1.42, 1.85; see Supplementary Figure S20) when both trauma and disease were measured using a prospective design.

On the other hand, when disease was measured prospectively, there was no significant association with abuse (OR = 1.27; 95% CI: 0.94, 1.72; see Supplementary Figure S21). All the results for prospective measures of both trauma and disease can be seen in Supplementary Table S17, while the results for prospective measures of disease only can be seen in Supplementary Table S18.

### Sensitivity analysis restricted to high-quality reviews

To assess the robustness of our finding, we conducted a sensitivity analysis restricted to reviews with an AMSTAR score ≥7. Overall, the primary class II associations reported above remained stable. Some reclassifications of the evidence levels occurred for specific associations, driven mainly by changes in the meta-analytic p-values after exclusion of lower quality reviews. These shifts were specific for ACE-disease pairings and do not affect the main conclusion of the study. Detailed results for the shifts of the sensitivity analysis are provided in the supplementary Table S19. The detailed AMSTAR scores per item for each review can be seen in Supplementary Table S20.

## Discussion

Overall, the results demonstrate a highly suggestive association (Class II) between any of the types of ACE and any of the types of non-mental medical disease included in our analysis, with an overall odds ratio of 1.57 (CI: 1.49–1.66), remaining highly suggestive but decreasing to an OR of 1.47 when diseases were assessed prospectively. The smaller odds ratios observed in prospective studies likely reflect reduced bias, leading therefore to more conservative estimates and a more realistic association. There is, specifically, highly suggestive evidence (Class II) for the association between abuse in general, sexual abuse, physical abuse or bullying and any non-mental medical disease, and also for the association between any type of ACE and headaches, IBS, diabetes, and CVD. There is also convincing evidence of an association between any type of ACE and CVD and between endocrine/nutritional/metabolic disease, when only prospective studies are taken into account, although, in this case, the magnitude of the association between ACE and CVD is small to modest. Regarding sex differences, females met the criteria for highly suggestive evidence (Class II) for any type of ACE and any physical disease, while males met suggestive evidence (Class III).

This work expands upon the existing evidence in terms of sample size and representativeness of the association between ACEs and all non-mental medical diseases by our systematic search including all relevant retrospective and prospective studies included in either systematic reviews or meta-analyses so far. Our findings are in line with previous research reinforcing the understanding that abuse is a clinically relevant factor associated with an increased risk of metabolic and cardiovascular conditions.^29^^,^^57^^,^^58^ It also is in line with the results of another large umbrella review showing that mental disorders contribute to a worse disease course in non-mental medical diseases.^7^ Therefore, in conjunction with previous studies, this umbrella review suggests that ACEs may be associated with higher rates of morbidity and mortality from chronic diseases and are consistently linked to the occurrence of some non-mental medical diseases, particularly metabolic and inflammatory diseases. However, the exact mechanism by which ACE can impact physical disease is poorly understood. First, ACE may have a negative impact on person's psychosocial development, affecting personal lifestyles and habits, which may lead to the adverse health behaviours including poor diet, decreased physical activity, smoking and excessive alcohol consumption that are known as modifiable factors to prevent metabolic complications.^59^ However, it is important to note that this study did not account for or control for cross-cultural variations, which should be considered when interpreting the findings. Secondly, mental health problems, especially affective, anxiety and depressive disorders, also impact the long-term health outcomes, emphasising the need to assess mental health in primary care. Thirdly, biological factors such as alterations in the autonomic nervous system, platelet receptors and function, coagulopathic factors, proinflammatory cytokines, endothelial function, neurohormonal factors,^60^ and genetics can also mediate and accelerate the onset of physical diseases.^61^

Regarding sex differences, a substantial body of research indicates that women exposed to ACEs are at a higher risk of developing adverse physical outcomes.^62^ Women may exhibit greater difficulty in habituating to stress responses, and the inability to adapt to repeated stressors is recognised as a risk factor for disease and diminished physical functioning.^63^ However, another important consideration is that female samples exhibit a higher prevalence of ACEs compared to male samples,^64^ and females face greater social vulnerability, with heightened exposure to environments conducive to violence and trauma during childhood. Consequently, it is essential to devise targeted protective strategies to mitigate the risk of traumatic experiences in this particularly vulnerable population.

These findings indicate that ACEs are a pervasive and potentially modifiable factor associated with both morbidity and mortality of many non-mental medical diseases. However, they should be interpreted as evidence of association rather than proof of causality, given the observational nature of the included studies, Although several associations are characterised by small to modest effect sizes, their high prevalence in the population may still translate into meaningful implications at the population level. Measures to identify at-risk individuals and treat ACE related symptoms can be incorporated into primary healthcare, and ACEs can be considered an additional vital sign that medical professionals should screen for.^65^ Therefore, we suggest specific interventions such systematic screening for ACEs in primary care, training healthcare professionals in trauma-informed care, integrated referral pathways to mental health services, preventive programs to promote resilience in high-risk families, and longitudinal monitoring to evaluate intervention effectiveness.

Implementing preventive measures and early interventions that address the long-term health effects of psychological trauma may contribute to enhancing overall well-being and alleviating the strain on healthcare systems. Given the widespread nature of childhood trauma and its enduring effects, clinicians may consider assessing a possible history of ACEs in patients presenting with physical conditions in primary medical care. Identifying abuse in clinical settings may facilitate earlier referrals to mental health professionals when appropriate. Early recognition of both the physical and psychological impacts of childhood trauma may help support more targeted and integrated care. Findings from this study underscore the potential relevance of early intervention and preventive strategies for at-risk groups, emphasising the lasting impact of early life stressors on physical health. From a policy perspective, these findings may support the need for a more integrated and transversal approach, including closer collaboration between primary care centres and mental health service. ACEs are strongly associated with headache, IBS, and diabetes, underscoring the importance of routine screening and integrated interventions that address both physical and mental health. Preventive strategies in schools and communities, coordinated care across health and social services, and research into resilience and effective interventions are essential to mitigate the long-term impact of early-life adversity.

Our subanalysis of prospective studies showed that these tended to exhibit less potential bias compared to retrospective studies in our umbrella review. This suggests that further methodologically robust prospective studies are needed to further clarify some of the associations found between ACE and medical disorders. Trauma assessment relied often on self-report measures, which can be affected by recall bias and the individual's subjective interpretation. However, subjective reports of trauma are more closely related to psychopathological outcomes than objective measures of trauma,^66^ but more evidence is needed to understand if the same pattern occurs with non-mental medical disease. Further research is also needed to elucidate the mechanisms behind the association of ACE and non-mental medical disease and to examine potential moderating factors such as genetic predispositions, environmental influences, and resilience factors that may influence the risk and the associations reported. A better understanding of the link between ACEs and non-mental medical disease may lead to new knowledge regarding pathophysiology and enhanced additional therapies for all patients.

Strengths of this umbrella review include its solid statistical methods, the large international and representative population, the clear results with a transversal impact on medicine, the differentiation between retrospective and prospective studies, and the inclusion of gender aspects. Limitations include that some associations did not reach convincing or highly suggestive evidence status because of the presence of heterogeneity in the sample as well as potential publication and excess significance biases. As specific analyses were extracted from each SR/MA, baseline characteristics (e.g., age and sex distribution) were not consistently available and could not be harmonised across studies. Therefore, heterogeneity in these characteristics may limit the extrapolation of the findings. Additionally, for some factors there was not a minimum of three studies, so these associations could not be analysed individually, which is a further limitation. Furthermore, trauma assessment methods vary between studies, with many relying on self-report measures, which can be affected by recall bias and the individual's subjective interpretation, and in some instances, the definition of ACEs is not clearly outlined in the methodologies of the included studies. Additionally, for common outcomes, OR may overestimate relative risks. Therefore, effect sizes should be interpreted cautiously. Last but not least, in the time between the search and finalising the paper, a brief search revealed that 275 new articles meeting the search string criteria in PubMed, Embase, and Web of Science had been published, which reduced to 194 once duplicates had been removed. Of these, 18 passed an initial title/abstract screen by one reviewer, of which 5 met criteria for inclusion in the review, meaning that although our results are robust and based on a large sample size, these papers have not been taken into account in our results.

## Contributors

Alicia Valiente-Gómez, as lead author, is the guarantor of the manuscript and shares first authorship with Joana Bucker. Adriane R. Rosa and Benedikt L. Amann, who had the idea of this work and supervised the development of this project, share senior authorship. Enric Vilajosana performed the statistical analysis. Daniel Guinart contributed to data extraction, analysis, interpretation, and draughting of the manuscript. Cristina de Córdoba, Carlos Campos-Rodríguez, Marta Fontana-McNally, Daniela Gatto and Helena Pardina-Torner contributed to data extraction, preparation of tables, and critical revision of the manuscript. Ana Moreno-Alcázar, Bridget Hogg, Adriane R. Rosa, and Joana Bucker contributed from the outset to study design, data extraction, analysis, and manuscript development. Joaquim Radua, Frank Padbergh, and Paolo Fusar-Poli provided expert advice and critical review. Bridget Hogg additionally contributed to overall study coordination, data extraction, analysis, and manuscript writing. All authors reviewed and approved the final version of the manuscript and Benedikt L. Amann was responsible for the decision to submit the manuscript. Alicia Valiente-Gómez, Joana Bücker and Bridget Hogg accessed and verified the underlying data.

## Data sharing statement

The dataset generated and analysed during the current study, including data extraction tables and supplementary analyses, is available in the Zenodo repository (https://doi.org/10.5281/zenodo.16921572).

## Declaration of interests

All authors have completed the Unified Competing Interest form and declare no support from any organisation for the submitted work; no financial relationships with any organisations that might have an interest in the submitted work in the previous three years, no other relationships or activities that could appear to have influenced the submitted work. Daniel Guinart reports grants or contracts from the European Commission and Instituto de Salud Carlos III; consulting fees from Angelini, Otsuka, Lundbeck, Teva, and Viatris; honoraria for lectures and presentations from Angelini, Otsuka, Lundbeck, Teva, and Viatris. Benedikt Lorenz Amann reports grants from the Horizon Europe MENTBEST project (n° 101080651), the PROSPERH project (n° 101137256), and the Spanish Ministry of Health/ISC-III (n° PI23/00072); and honoraria for lectures and presentations from the Spanish Psychiatric Association, EMDR Spain, and EMDR Europe. Frank Padbergh reports honoraria for lectures on child maltreatment and the Cognitive Behavioral Analysis System of Psychotherapy (CBASP) from AWIP Ulm and Kirinus Munich, and a keynote lecture for EMDR Europe. Carlos Campos Rodríguez is supported by a research contract under HORIZON-HLTH-2023-ENVHLTH-02 (ID: 101137256). Marta Fontana-McNally is supported by a PFIS grant from the Instituto de Salud Carlos III. Paolo Fusar-Poli has received payments or honoraria from Recordati, Lundbeck, Angelini, Menarini, Sunovion, Boehringer Ingelheim, Proxymm Science, Otsuka, Gedeon Richter, CurieBio outside the current study. The rest of the authors have no conflicts of interest declared.
