# Supplementary material for: Adverse childhood experiences and risk of non-mental medical diseases in adulthood: an umbrella review
Source: eClinicalMedicine. 2026 May 22;96:103987. doi: 10.1016/j.eclinm.2026.103987 (PMC13223999; doi:10.1016/j.eclinm.2026.103987)

Supplementary Information, Tables, and Figures

Supplementary Information 1 Search Strategy

PubMed – Search Strategy

(((Stress Disorders, Traumatic[mh] OR Traumatic Stress Disorder*[tiab] OR Psychological Trauma*[tiab] OR Psychotrauma*[tiab] OR Sexual Trauma*[tiab] OR Sexual Abuse*[tiab] OR Post-Traumatic Stress Disorder*[tiab] OR Post-Traumatic Neuros*[tiab] OR PTSD[tiab] OR Posttraumatic Neuros*[tiab] OR Moral Injur*[tiab] OR Acute Stress Disorder*[tiab] OR Stressful event*[tiab] OR Traumatic event*[tiab] OR Elder Abuse*[tiab] OR Aged Abuse*[tiab] OR Elder Neglect*[tiab] OR Elderly Abuse*[tiab] OR Senior Abuse*[tiab] OR Elder Mistreat*[tiab] OR Elder Maltreat*[tiab] OR Spouse Abuse*[tiab] OR Spousal Abuse*[tiab] OR Partner Abuse*[tiab] OR Wife Abuse*[tiab] OR Partner Abuse*[tiab] OR Emotional Abuse[mh] OR Emotional abuse*[tiab] OR Psychological Abuse*[tiab] OR Emotional neglect*[tiab] OR Physical neglect*[tiab] OR Bullying[mh] OR Bullying[tiab] OR Cyberbullying[tiab] OR Physical Abuse*[tiab] OR Physical Maltreatment*[tiab]) AND (Child[mh] OR Adolescent[mh] OR child[tiab] OR children[tiab] OR childhood[tiab] OR adolescen*[tiab] OR paediatric*[tiab] OR pediatric*[tiab] OR teen*[tiab])) OR Adverse Childhood Experiences[mh] OR Adverse Childhood Experience*[tiab] OR Adverse Child Experience*[tiab] OR
Adverse Children Experience*[tiab] OR Childhood Trauma*[tiab] OR Traumatic Childhood Experience*[tiab] OR Adolescent Trauma*[tiab] OR Early Life Stress*[tiab] OR Child Abuse*[tiab] OR Child Mistreat*[tiab] OR Child Maltreat*[tiab] OR Child Neglect*[tiab] OR Child Molest*[tiab] OR Battered Child Syndrome*[tiab])

AND

(Somatic*[tiab] OR Clinical disorder*[tiab] OR Clinical condition*[tiab] OR Medical disorder*[tiab] OR Medical condition*[tiab] OR Clinical health*[tiab] OR Underlying disorder*[tiab] OR Underlying condition*[tiab] OR Comorbidity[mh] OR Comorbid*[tiab] OR Multimorbid*[tiab] OR Additional disorder*[tiab] OR Additional condition*[tiab] OR Pathologic Processes[mh] OR Patholog*[tiab] OR Physical health*[tiab] OR Physical condition*[tiab] OR Health outcome*[tiab] OR Nervous System Diseases[mh] OR Nervous System Disease*[tiab] OR Neurologic Disorder*[tiab] OR Neurological Disorder*[tiab] OR Nervous System Disorder*[tiab] OR Neoplasms[mh] OR Neoplas*[tiab] OR Tumor*[tiab] OR Tumour*[tiab] OR Cancer*[tiab] OR Malignan*[tiab] OR Oncolog*[tiab] OR Urologic Diseases[mh] OR Urologic Disease*[tiab] OR Urological Disease*[tiab] OR Urinary Tract Disease*[tiab] OR Kidney Disease*[tiab] OR Cardiovascular Diseases[mh] OR Cardiovascular Disease*[tiab] OR Heart Disease*[tiab] OR Cardiac Disease*[tiab] OR Cardiac Disorder*[tiab] OR Heart Disorder*[tiab] OR Cardiac Event*[tiab] OR Immune System Diseases[mh] OR Immune System Disease*[tiab] OR Immunologic Disease*[tiab] OR Immunological Disease*[tiab] OR Immune Disease*[tiab] OR Immune Disorder*[tiab] OR Immune System Disorder*[tiab] OR Digestive System Diseases[mh] OR Digestive System Disease*[tiab] OR Digestive System Disorder*[tiab] OR Hepatobiliary Disorder*[tiab] OR Hepatobiliary Disease*[tiab] OR Obesity[mh] OR Obesity[tiab] OR Obese[tiab] OR Skin Diseases[mh] OR Skin Disease*[tiab] OR Dermatos*[tiab] OR Skin Disorder*[tiab] OR Subcutaneous Tissue Disorder*[tiab] OR Endocrine System Diseases[mh] OR Endocrine System Disease*[tiab] OR Endocrine Disease*[tiab])

AND

((systematic*[ti] AND review[ti]) OR (Systematic overview*[ti] OR Cochrane review*[ti] OR systemic review*[ti] OR scoping review[ti] OR scoping literature review[ti] OR mapping review[ti] OR Umbrella review*[ti] OR review of reviews[ti] OR overview of reviews[ti] OR meta-review[ti] OR integrative review[ti] OR integrated review[ti] OR integrative overview[ti] OR meta-analyses[ti] OR metaanalyses[ti] OR metaanalysis[ti] OR meta-analysis[ti] OR metanalyses[ti] OR metanalysis[ti] OR meta-analytic review[ti] OR meta-analytical review[ti] OR meta-analysis[pt]) NOT (letter[pt] OR editorial[pt] OR comment[pt] OR case reports[pt] OR historical article[pt] OR report[ti] OR protocol[ti] OR protocols[ti] OR withdrawn[ti] OR retraction of publication[pt] OR retraction of publication as topic[mesh] OR retracted publication[pt] OR reply[ti] OR published erratum[pt]))

Embase – Search Strategy

((('posttraumatic stress disorder'/de OR 'sexual trauma'/exp OR 'acute stress disorder'/exp OR 'psychotrauma'/de OR 'sexual abuse'/exp OR 'moral injury'/exp OR 'stressful life event'/exp OR 'elder abuse'/exp OR 'battered woman'/exp OR 'family violence'/exp OR 'partner violence'/exp OR 'emotional abuse'/exp OR 'bullying'/exp OR 'traumatic stress disorder*':ti,ab,kw OR 'psychological trauma*':ti,ab,kw OR 'psychotruma*':ti,ab,kw OR 'sexual trauma*':ti,ab,kw OR 'sexual abuse*':ti,ab,kw OR 'post-traumatic stress disorder*':ti,ab,kw OR 'post-traumatic neuros*':ti,ab,kw OR 'ptsd':ti,ab,kw OR 'posttraumatic neuros*':ti,ab,kw OR 'moral injur*':ti,ab,kw OR 'acute stress disorder*':ti,ab,kw OR 'stressful event*':ti,ab,kw OR 'traumatic event*':ti,ab,kw OR 'elder abuse*':ti,ab,kw OR 'aged abuse*':ti,ab,kw OR 'elder neglect*':ti,ab,kw OR 'elderly abuse*':ti,ab,kw OR 'senior abuse*':ti,ab,kw OR 'elder mistreat*':ti,ab,kw OR 'elder maltreat*':ti,ab,kw OR 'spouse abuse*':ti,ab,kw OR 'spousal abuse*':ti,ab,kw OR 'wife abuse*':ti,ab,kw OR 'partner abuse*':ti,ab,kw OR 'emotional abuse*':ti,ab,kw OR 'psychological abuse*':ti,ab,kw OR 'emotional neglect*':ti,ab,kw OR 'physical neglect*':ti,ab,kw OR 'bullying':ti,ab,kw OR 'cyberbullying':ti,ab,kw OR 'physical abuse*':ti,ab,kw OR 'physical maltreatment*':ti,ab,kw) AND ('child'/exp OR 'adolescent'/de OR child:ti,ab,kw OR children:ti,ab,kw OR childhood:ti,ab,kw OR adolescen*:ti,ab,kw OR paediatric*:ti,ab,kw OR pediatric*:ti,ab,kw OR teen*:ti,ab,kw)) OR ('childhood adversity'/de OR 'child abuse'/exp OR 'adverse childhood experience*':ti,ab,kw OR 'adverse child experience*':ti,ab,kw OR 'adverse children experience*':ti,ab,kw OR 'childhood trauma*':ti,ab,kw OR 'traumatic childhood experience*':ti,ab,kw OR 'adolescent trauma*':ti,ab,kw OR 'early life stress*':ti,ab,kw OR 'child abuse*':ti,ab,kw OR 'child mistreat*':ti,ab,kw OR 'child maltreat*':ti,ab,kw OR 'child neglect*':ti,ab,kw OR 'child molest*':ti,ab,kw OR 'battered child syndrome*':ti,ab,kw))

AND

('Somatic*':ti,ab,kw OR 'Clinical disorder*':ti,ab,kw OR 'Clinical condition*':ti,ab,kw OR 'Medical disorder*':ti,ab,kw OR 'Medical condition*':ti,ab,kw OR 'Clinical health*':ti,ab,kw OR 'Underlying disorder*':ti,ab,kw OR 'Underlying condition*':ti,ab,kw OR 'comorbidity'/de OR 'Comorbid*':ti,ab,kw OR 'Multimorbid*':ti,ab,kw OR 'physical disease'/de OR 'Additional disorder*':ti,ab,kw OR 'Additional condition*':ti,ab,kw OR 'Patholog*':ti,ab,kw OR 'Physical health*':ti,ab,kw OR 'Physical condition*':ti,ab,kw OR 'Health outcome*':ti,ab,kw OR 'neurologic disease'/exp OR 'Nervous System Disease*':ti,ab,kw OR 'Neurologic Disorder*':ti,ab,kw OR 'Neurological Disorder*':ti,ab,kw OR 'Nervous System Disorder*':ti,ab,kw OR 'neoplasm'/exp OR 'Neoplas*':ti,ab,kw OR 'Tumor*':ti,ab,kw OR 'Tumour*':ti,ab,kw OR 'Cancer*':ti,ab,kw OR 'Malignan*':ti,ab,kw OR 'Oncolog*':ti,ab,kw OR 'urinary tract disease'/exp OR 'Urologic Disease*':ti,ab,kw OR 'Urological Disease*':ti,ab,kw OR 'Urinary Tract Disease*':ti,ab,kw OR 'Kidney Disease*':ti,ab,kw OR 'cardiovascular disease'/exp OR 'Cardiovascular Disease*':ti,ab,kw OR 'Heart Disease*':ti,ab,kw OR 'Cardiac Disease*':ti,ab,kw OR 'Cardiac Disorder*':ti,ab,kw OR 'Heart Disorder*':ti,ab,kw OR 'Cardiac Event*':ti,ab,kw OR 'immunopathology'/exp OR 'Immune System Disease*':ti,ab,kw OR 'Immunologic Disease*':ti,ab,kw OR 'Immunological Disease*':ti,ab,kw OR 'Immune Disease*':ti,ab,kw OR 'Immune Disorder*':ti,ab,kw OR 'Immune System Disorder*':ti,ab,kw OR 'digestive system disease'/exp OR 'Digestive System Disease*':ti,ab,kw OR 'Digestive System Disorder*':ti,ab,kw OR 'Hepatobiliary Disorder*':ti,ab,kw OR 'Hepatobiliary Disease*':ti,ab,kw OR 'obesity'/exp OR 'Obesity':ti,ab,kw OR 'Obese':ti,ab,kw OR 'skin disease'/exp OR 'Skin Disease*':ti,ab,kw OR 'Dermatos*':ti,ab,kw OR 'Skin Disorder*':ti,ab,kw OR 'Subcutaneous Tissue Disorder*':ti,ab,kw OR 'endocrine disease'/exp OR 'Endocrine System Disease*':ti,ab,kw OR 'Endocrine Disease*':ti,ab,kw)

AND

(((systematic*:ti AND review:ti) OR ('Systematic overview*':ti OR 'Cochrane review*':ti OR 'systemic review*':ti OR 'scoping review':ti OR 'scoping literature review':ti OR 'mapping review':ti OR 'Umbrella review*':ti OR 'review of reviews':ti OR 'overview of reviews':ti OR 'meta-review':ti OR 'integrative review':ti OR 'integrated review':ti OR 'integrative overview':ti OR 'meta-analyses':ti OR 'metaanalyses':ti OR

'metaanalysis':ti OR 'meta-analysis':ti OR 'metanalyses':ti OR 'metanalysis':ti OR 'meta-analytic review':ti OR 'meta-analytical review':ti OR 'meta-analysis'/it)) NOT ('chapter'/it OR 'editorial'/it OR 'erratum'/it OR 'letter'/it OR 'note'/it OR 'short survey'/it OR 'tombstone'/it))

AND

([embase]/lim NOT ([embase]/lim AND [medline]/lim))

Web of Science Search Strategy

((TS=("Traumatic Stress Disorder*" OR "Psychological Trauma*" OR "Psychotruma*" OR "Sexual Trauma*" OR "Sexual Abuse*" OR "Post-Traumatic Stress Disorder*" OR "Post-Traumatic Neuros*" OR "PTSD" OR "Posttraumatic Neuros*" OR "Moral Injur*" OR "Acute Stress Disorder*" OR "Stressful event*" OR "Traumatic event*" OR "Elder Abuse*" OR "Aged Abuse*" OR "Elder Neglect*" OR "Elderly Abuse*" OR "Senior Abuse*" OR "Elder Mistreat*" OR "Elder Maltreat*" OR "Spouse Abuse*" OR "Spousal Abuse*" OR "Partner Abuse*" OR "Wife Abuse*" OR "Partner Abuse*" OR "Emotional abuse*" OR "Psychological Abuse*" OR "Emotional neglect*" OR "Physical neglect*" OR "Bullying" OR "Cyberbullying" OR "Physical Abuse*" OR "Physical Maltreatment*")

AND

TS=("child" OR "children" OR "childhood" OR "adolescen*" OR "paediatric*" OR "pediatric*" OR "teen*"))

OR

TS=("Adverse Childhood Experience*" OR "Adverse Child Experience*" OR "Adverse Children Experience*" OR "Childhood Trauma*" OR "Traumatic Childhood Experience*" OR "Adolescent Trauma*" OR "Early Life Stress*" OR "Child Abuse*" OR "Child Mistreat*" OR "Child Maltreat*" OR "Child Neglect*" OR "Child Molest*" OR "Battered Child Syndrome*"))

AND

TS=("Somatic*" OR "Clinical disorder*" OR "Clinical condition*" OR "Medical disorder*" OR "Medical condition*" OR "Clinical health*" OR "Underlying disorder*" OR "Underlying condition*" OR "Comorbid*" OR "Multimorbid*" OR "Additional disorder*" OR "Additional condition*" OR "Patholog*" OR "Physical health*" OR "Physical condition*" OR "Health outcome*" OR "Nervous System Disease*" OR "Neurologic Disorder*" OR "Neurological Disorder*" OR "Nervous System Disorder*" OR "Neoplas*" OR "Tumor*" OR "Tumour*" OR "Cancer*" OR "Malignan*" OR "Oncolog*" OR "Urologic Disease*" OR "Urological Disease*" OR "Urinary Tract Disease*" OR "Kidney Disease*" OR "Cardiovascular Disease*" OR "Heart Disease*" OR "Cardiac Disease*" OR "Cardiac Disorder*" OR "Heart Disorder*" OR "Cardiac Event*" OR "Immune System Disease*" OR "Immunologic Disease*" OR "Immunological Disease*" OR "Immune Disease*" OR "Immune Disorder*" OR "Immune System Disorder*" OR "Digestive System Disease*" OR "Digestive System Disorder*" OR "Hepatobiliary Disorder*" OR "Hepatobiliary Disease*" OR "Obesity" OR "Obese" OR "Skin Disease*" OR "Dermatos*" OR "Skin Disorder*" OR "Subcutaneous Tissue Disorder*" OR "Endocrine System Disease*" OR "Endocrine Disease*")

AND

(TI=(systematic* AND review)

OR

TI=("Systematic overview*" OR "Cochrane review*" OR "systemic review*" OR "scoping review" OR

"scoping literature review" OR "mapping review" OR "Umbrella review*" OR "review of reviews" OR "overview of reviews" OR "meta-review" OR "integrative review" OR "integrated review" OR "integrative overview" OR "meta-analyses" OR "metaanalyses" OR "metaanalysis" OR "meta-analysis" OR "metanalyses" OR "metanalysis" OR "meta-analytic review" OR "meta-analytical review"))

Supplementary Information 2 List of excluded articles at full-text screening stage

Reasons for exclusion	First author	Year
1 - not systematic review or meta-analysis	Iloson	2021
	Boch	2018
	Carr	2020
	Lee	2022
	Hadwen	2022
	Bussieres	2020
	Chandan	2019
	Marques	2022
	Entringer	2016
	Scherbakov	2024
	Mellen	2024
	Niedenfuehr	2023
	Ploesser	2024
	Bochicchio	2024
	Perez	2024
	Willoughby	2024
	Jacobs	2024
	Bialy	2024
	Shanahan	2024
	Grant	2023
	Haczkewicz	2024
	Qamar	2024
	Matson	2024
	Deglon	2023
	Thornton	2025
	Kandlur	2023
	Lee	2024
	Zhou	2023
	Cooper	2025
	Reid	2023
2 - no somatic disorder as result of trauma	Carlos Jefferson do Nascimento	2019
	Pfaff	2021
	van Geel	2016
	Cheng	2022
	Snast	2018
	Steine	2012
	Pinquart	2020
	Vadukapuram	2022
	Ba	2017
	Peer	2020
	Arriola	2005
	Yu	2022
	Marin	2021
	Maccarrone	2021
	Solmi	2021
	Sartori	2023
	Maguire	2013
	Panisch	2020
	An	2019
	Mohan	2022
	Potzauf	2022

	Vadukapuram	2021
	Spies	2020
	Turner	2019
	Cartagena	2018

	Philips	2012
	Sonneveld	2011
	Watanabe	2023
	Helmy	2024
	Zhang	2024
	Conti	2023
	Otterman	2024
	Buecker	2024
	Ciocca	2023
	Smith	2023
	Maayan	2024
	Bower	2023
	Gao	2024
	Witteveen	2023
	Mamun	2023
	Mhlongo	2023
	Sarker	2023
	Papola	2024
	Girgla	2023
	Dionisio-García	2023
	Melamed	2024
	Tareke	2023
	Lin	2024
	Lohmann	2024
	Liu	2024
	Shao	2024
	Jural	2024
	Dowling	2023
	Racine	2023
	Xie	2024
	Adjimi Nyemgah	2024
	Chang	2024
	Collier	2023
	Schmidt	2024
	Benedict	2025
	Li	2024
	Spencer	2024
	Zou	2024
	Bailey	2024
	Wilson	2025
	Majumder	2024
	Chia	2025
	Zu	2025
	Nocerino	2024
	Quodling	2024
	Thiemann	2024
	Onyeama	2024
	Rodríguez	2024
	Predescu	2024
	Spínola	2024
	di Giacomo	2024

	El-Haj-Mohamad	2023
	Nasiri	2023
	Buchanan	2023
	Lloyd	2023
	AlHamawi	2023
	Meyer	2023
	Law	2023
	Makkar	2023
	Richardson	2023
	Aljadani	2023
	Jourdan	2023
	Mishra	2023
	Dibben	2023
	Senberg	2023
	Wang	2023
	Radtke	2024
	Yuan	2023
	Zhu	2023
	Ko	2024
	Deol	2023
	Lombardo	2024
	Uthayakumar	2023
	Shorey	2023
	Davis	2023
	Oppenheim	2024
	Sandnes	2024
	Vyas	2023
	Zagaria	2024
	Dien Esquivel	2024
	Vidal	2024
	Tseng	2024
	Mthiyane	2023
	Han	2023
	Bacaro	2023
	Williams	2024
	de Pellegars	2024
	Low	2024
	Musindo	2023
	Chu	2024
	Bruzelius	2024
	Lazzari	2024
	Mohammed	2024
	Moore	2024
	Counts	2024
	Lima Constantino	2025
	Yan	2025
	Hong	2025
	Everri	2024
	Nishida	2024
	Son	2024
	Culos	2024
	Lu	2024
	Abate	2024
	Kirakosyan	2024
	Al Hajj	2023

	Mohammed	2023
	Alameda	2023
	Torbahn	2023
	May	2023
	Loizou	2024
3 - association between trauma and somatic disorder not studies	McDonnell	2018
	Saygideger	2022
	Steinig	2012
	Cunha	2024
	Clasby	2023
	McBain	2023
	van Dijl	2024
	Siahaan	2023
	Meurillon	2024
	Di Fazio	2023
	Chen	2023
	Ozdemir Koyu	2023
	Zhan	2024
	Paterson	2023
	Boakye	2024
	Hutchinson	2024
	Placek	2024
	Knutzen	2024
	Shenoy	2024
4 - no control group (either for trauma and/or somatic disorder)	Aygin	2019
	Midei	2011
	Ottisova	2016
6 - insufficient data for our review	Saulnier	2015
	Carr	2020
	Jakubowski	2017
	Malwane	2022
	Kacel	2016
	Poliakin	2018
7 - wrong population	Virgilio	2021
	Sheikhnezhad	2023
	Grafft	2024
	Mundorf	2024
	Alves	2024
	Wei	2024

Supplementary Information 3. PRISMA, PRIOR and MOOSE checklists.

MOOSE (Meta-analyses Of Observational Studies in Epidemiology) Checklist

A reporting checklist for Authors, Editors, and Reviewers of Meta-analyses of Observational Studies. You must report the page number in your manuscript where you consider each of the items listed in this checklist. If you have not included this information, either revise your manuscript accordingly before submitting or note N/A.

Reporting Criteria	Reported (Yes/No)	Reported on Page No.
**Reporting of Background**		
Problem definition	Yes	5
Hypothesis statement	Yes	5
Description of Study Outcome(s)	Yes	5
Type of exposure or intervention used	Yes	5
Type of study design used	Yes	5
Study population	Yes	5
**Reporting of Search Strategy**		
Qualifications of searchers (eg, librarians and investigators)	Yes	5
Search strategy, including time period included in the synthesis and keywords	Yes	5,6
Effort to include all available studies, including contact with authors	Yes	6
Databases and registries searched	Yes	5
Search software used, name and version, including special features used (eg, explosion)	Yes	5
Use of hand searching (eg, reference lists of obtained articles)	Yes	6, 8
List of citations located and those excluded, including justification	Yes	6, 8
Method for addressing articles published in languages other than English	Yes	5
Method of handling abstracts and unpublished studies	Yes	5
Description of any contact with authors	Yes	6
**Reporting of Methods**		
Description of relevance or appropriateness of studies assembled for assessing the hypothesis to be tested	Yes	6
Rationale for the selection and coding of data (eg, sound clinical principles or convenience)	Yes	6
Documentation of how data were classified and coded (eg, multiple raters, blinding, and interrater reliability)	Yes	6
Assessment of confounding (eg, comparability of cases and controls in studies where appropriate	Yes	6

Reporting Criteria	Reported (Yes/No)	Reported on Page No.
Assessment of study quality, including blinding of quality assessors; stratification or regression on possible predictors of study results	Yes	6
Assessment of heterogeneity	Yes	6
Description of statistical methods (eg, complete description of fixed or random effects models, justification of whether the chosen models account for predictors of study results, dose-response models, or cumulative meta-analysis) in sufficient detail to be replicated	Yes	6,7
Provision of appropriate tables and graphics	Yes	7
**Reporting of Results**		
Table giving descriptive information for each study included	Yes	8
Results of sensitivity testing (eg, subgroup analysis)	Yes	8-10
Indication of statistical uncertainty of findings	Yes	8
**Reporting of Discussion**		
Quantitative assessment of bias (eg, publication bias)	Yes	10
Justification for exclusion (eg, exclusion of non–English-language citations)	Yes	6
Assessment of quality of included studies	Yes	8
**Reporting of Conclusions**		
Consideration of alternative explanations for observed results	Yes	11
Generalization of the conclusions (ie, appropriate for the data presented and within the domain of the literature review)	Yes	10-12
Guidelines for future research	Yes	11
Disclosure of funding source	Yes	13

Once you have completed this checklist, please save a copy and upload it as part of your submission. DO NOT include this checklist as part of the main manuscript document. It must be uploaded as a separate file.

PRIOR Checklist

(Gates M, Gates A, Pieper D, et al. Reporting guideline for overviews of reviews of healthcare interventions: development of the PRIOR statement. *BMJ* 2022;378:e070849. doi:10.1136/bmj-2022-070849.)

**Section** Topic	#	**Item**	**Location reported**
**TITLE**			
Title	1	Identify the report as an overview of reviews.	1
**ABSTRACT**			
Abstract	2	Provide a comprehensive and accurate summary of the purpose, methods, and results of the overview of reviews.	2
**INTRODUCTION**			
Rationale	3	Describe the rationale for conducting the overview of reviews in the context of existing knowledge.	5
Objectives	4	Provide an explicit statement of the objective(s) or question(s) addressed by the overview of reviews.	5
**METHODS**			
Eligibility criteria	5a	Specify the inclusion and exclusion criteria for the overview of reviews. If supplemental primary studies were included, this should be stated, with a rationale.	6, 7
	5b	Specify the definition of 'systematic review' as used in the inclusion criteria for the overview of reviews.	6, 7
Information sources	6	Specify all databases, registers, websites, organizations, reference lists, and other sources searched or consulted to identify systematic reviews and supplemental primary studies (if included). Specify the date when each source was last searched or consulted.	5
Search strategy	7	Present the full search strategies for all databases, registers and websites, such that they could be reproduced. Describe any search filters and limits applied.	5
Selection process	8a	Describe the methods used to decide whether a systematic review or supplemental primary study (if included) met the inclusion criteria of the overview of reviews.	5, 6
	8b	Describe how overlap in the populations, interventions, comparators, and/or outcomes of systematic reviews was identified and managed during study selection.	6
Data collection process	9a	Describe the methods used to collect data from reports.	6
	9b	If applicable, describe the methods used to identify and manage primary study overlap at the level of the comparison and outcome during data collection. For each outcome, specify the method used to illustrate and/or quantify the degree of primary study overlap across systematic reviews.	6
	9c	If applicable, specify the methods used to manage discrepant data across systematic reviews during data collection.	6
Data items	10	List and define all variables and outcomes for which data were sought. Describe any assumptions made and/or measures taken to identify and clarify missing or unclear information.	6
Risk of bias assessment	11a	Describe the methods used to *assess* risk of bias or methodological quality of the included systematic reviews.	6
	11b	Describe the methods used to *collect* data on (from the systematic reviews) and/or *assess* the risk of bias of the primary studies included in the systematic reviews. Provide a justification for instances where flawed, incomplete, or missing assessments are identified but not re-assessed.	6
	11c	Describe the methods used to *assess* the risk of bias of supplemental primary studies (if included).	6
Synthesis methods	12a	Describe the methods used to summarize or synthesize results and provide a rationale for the choice(s).	6
	12b	Describe any methods used to explore possible causes of heterogeneity among results.	6
	12c	Describe any sensitivity analyses conducted to assess the robustness of the synthesized results.	6
Reporting bias assessment	13	Describe the methods used to *collect* data on (from the systematic reviews) and/or *assess* the risk of bias due to missing results in a summary or synthesis (arising from reporting biases at the levels of the systematic reviews, primary studies, and supplemental primary studies, if included).	6

Certainty assessment	14	Describe the methods used to *collect* data on (from the systematic reviews) and/or *assess* certainty (or confidence) in the body of evidence for an outcome.	6
**RESULTS**			
Systematic review and supplemental primary study selection	15a	Describe the results of the search and selection process, including the number of records screened, assessed for eligibility, and included in the overview of reviews, ideally with a flow diagram.	8
	15b	Provide a list of studies that might appear to meet the inclusion criteria, but were excluded, with the main reason for exclusion.	6
**Section** Topic	**#**	**Item**	**Location reported**
Characteristics of systematic reviews and supplemental primary studies	16	Cite each included systematic review and supplemental primary study (if included) and present its characteristics.	8
Primary study overlap	17	Describe the extent of primary study overlap across the included systematic reviews.	7
Risk of bias in systematic reviews, primary studies, and supplemental primary studies	18a	Present assessments of risk of bias or methodological quality for each included systematic review.	8
	18b	Present assessments (*collected* from systematic reviews or *assessed* anew) of the risk of bias of the primary studies included in the systematic reviews.	8
	18c	Present assessments of the risk of bias of supplemental primary studies (if included).	8
Summary or synthesis of results	19a	For all outcomes, summarize the evidence from the systematic reviews and supplemental primary studies (if included). If meta-analyses were done, present for each the summary estimate and its precision and measures of statistical heterogeneity. If comparing groups, describe the direction of the effect.	8
	19b	If meta-analyses were done, present results of all investigations of possible causes of heterogeneity.	8
	19c	If meta-analyses were done, present results of all sensitivity analyses conducted to assess the robustness of synthesized results.	8
Reporting biases	20	Present assessments (*collected* from systematic reviews and/or *assessed* anew) of the risk of bias due to missing primary studies, analyses, or results in a summary or synthesis (arising from reporting biases at the levels of the systematic reviews, primary studies, and supplemental primary studies, if included) for each summary or synthesis assessed.	8
Certainty of evidence	21	Present assessments (*collected* or *assessed* anew) of certainty (or confidence) in the body of evidence for each outcome.	8
**DISCUSSION**			
Discussion	22a	Summarize the main findings, including any discrepancies in findings across the included systematic reviews and supplemental primary studies (if included).	10
	22b	Provide a general interpretation of the results in the context of other evidence.	10
	22c	Discuss any limitations of the evidence from systematic reviews, their primary studies, and supplemental primary studies (if included) included in the overview of reviews. Discuss any limitations of the overview of reviews methods used.	11
	22d	Discuss implications for practice, policy, and future research (both systematic reviews and primary research). Consider the relevance of the findings to the end users of the overview of reviews, e.g., healthcare providers, policymakers, patients, among others.	11
**OTHER INFORMATION**			
Registration and protocol	23a	Provide registration information for the overview of reviews, including register name and registration number, or state that the overview of reviews was not registered.	5
	23b	Indicate where the overview of reviews protocol can be accessed, or state that a protocol was not prepared.	5
	23c	Describe and explain any amendments to information provided at registration or in the protocol. Indicate the stage of the overview of reviews at which amendments were made.	5
Support	24	Describe sources of financial or non-financial support for the overview of reviews, and the role of the funders or sponsors in the overview of reviews.	13

Competing interests	25	Declare any competing interests of the overview of reviews' authors.	13
Author information	26a	Provide contact information for the corresponding author.	1
	26b	Describe the contributions of individual authors and identify the guarantor of the overview of reviews.	12
Availability of data and other materials	27	Report which of the following are available, where they can be found, and under which conditions they may be accessed: template data collection forms; data collected from included systematic reviews and supplemental primary studies; analytic code; any other materials used in the overview of reviews.	13

PRISMA 2020 Checklist

Section and Topic	Item #	Checklist item	Location where item is reported
**TITLE**			
Title	1	Identify the report as a systematic review.	Title, Page 1
**ABSTRACT**			
Abstract	2	See the PRISMA 2020 for Abstracts checklist.	Summary, Page 2
**INTRODUCTION**			
Rationale	3	Describe the rationale for the review in the context of existing knowledge.	Introduction, Page 5
Objectives	4	Provide an explicit statement of the objective(s) or question(s) the review addresses.	Introduction, Page 5
**METHODS**			
Eligibility criteria	5	Specify the inclusion and exclusion criteria for the review and how studies were grouped for the syntheses.	Methods, Page 5
Information sources	6	Specify all databases, registers, websites, organisations, reference lists and other sources searched or consulted to identify studies. Specify the date when each source was last searched or consulted.	Methods, Page 5
Search strategy	7	Present the full search strategies for all databases, registers and websites, including any filters and limits used.	Supplementary information, Page 2
Selection process	8	Specify the methods used to decide whether a study met the inclusion criteria of the review, including how many reviewers screened each record and each report retrieved, whether they worked independently, and if applicable, details of automation tools used in the process.	Methods, Page 6
Data collection process	9	Specify the methods used to collect data from reports, including how many reviewers collected data from each report, whether they worked independently, any processes for obtaining or confirming data from study investigators, and if applicable, details of automation tools used in the process.	Methods, Page 6
Data items	10a	List and define all outcomes for which data were sought. Specify whether all results that were compatible with each outcome domain in each study were sought (e.g. for all measures, time points, analyses), and if not, the methods used to decide which results to collect.	Methods, Page 6
	10b	List and define all other variables for which data were sought (e.g. participant and intervention characteristics, funding sources). Describe any assumptions made about any missing or unclear information.	Methods, Page 6
Study risk of bias assessment	11	Specify the methods used to assess risk of bias in the included studies, including details of the tool(s) used, how many reviewers assessed each study and whether they worked independently, and if applicable, details of automation tools used in the process.	Methods, Page 7
Effect measures	12	Specify for each outcome the effect measure(s) (e.g. risk ratio, mean difference) used in the synthesis or presentation of results.	Methods, Page 6
Synthesis methods	13a	Describe the processes used to decide which studies were eligible for each synthesis (e.g. tabulating the study intervention characteristics and comparing against the planned groups for each synthesis (item #5)).	Methods, Page 6
	13b	Describe any methods required to prepare the data for presentation or synthesis, such as handling of missing summary statistics, or data conversions.	Methods, Page 6
	13c	Describe any methods used to tabulate or visually display results of individual studies and syntheses.	Methods, Page 6
	13d	Describe any methods used to synthesize results and provide a rationale for the choice(s). If meta-analysis was performed, describe the	Methods Page

Section and Topic	Item #	Checklist item	Location where item is reported
		model(s), method(s) to identify the presence and extent of statistical heterogeneity, and software package(s) used.	6
	13e	Describe any methods used to explore possible causes of heterogeneity among study results (e.g. subgroup analysis, meta-regression).	Methods, Page 6
	13f	Describe any sensitivity analyses conducted to assess robustness of the synthesized results.	Methods, Page 6
Reporting bias assessment	14	Describe any methods used to assess risk of bias due to missing results in a synthesis (arising from reporting biases).	Methods, Page 6
Certainty assessment	15	Describe any methods used to assess certainty (or confidence) in the body of evidence for an outcome.	Methods, Page 7
**RESULTS**			
Study selection	16a	Describe the results of the search and selection process, from the number of records identified in the search to the number of studies included in the review, ideally using a flow diagram.	Results, Page 7 and Figure 1
	16b	Cite studies that might appear to meet the inclusion criteria, but which were excluded, and explain why they were excluded.	Supplementary material, Page 6
Study characteristics	17	Cite each included study and present its characteristics.	Table 2. Page 8.
Risk of bias in studies	18	Present assessments of risk of bias for each included study.	Table 2. Page 8.
Results of individual studies	19	For all outcomes, present, for each study: (a) summary statistics for each group (where appropriate) and (b) an effect estimate and its precision (e.g. confidence/credible interval), ideally using structured tables or plots.	Results, Pages 7-10, Figures 2 and 3 and supplementary material
Results of syntheses	20a	For each synthesis, briefly summarise the characteristics and risk of bias among contributing studies.	Results, Pages 7-10, and supplementary material
	20b	Present results of all statistical syntheses conducted. If meta-analysis was done, present for each the summary estimate and its precision (e.g. confidence/credible interval) and measures of statistical heterogeneity. If comparing groups, describe the direction of the effect.	Results, Pages 7-10, Figures 2 and 3 and supplementary material
	20c	Present results of all investigations of possible causes of heterogeneity among study results.	Results, Page 7-10, and supplementary material
	20d	Present results of all sensitivity analyses conducted to assess the robustness of the synthesized results.	Results, Pages 7-10, and supplementary

PRISMA 2020 Checklist

Section and Topic	Item #	Checklist item	Location where item is reported
			material
Reporting biases	21	Present assessments of risk of bias due to missing results (arising from reporting biases) for each synthesis assessed.	Results, Pages 7-10, and supplementary material
Certainty of evidence	22	Present assessments of certainty (or confidence) in the body of evidence for each outcome assessed.	Results, Pages 7-10, and supplementary material
**DISCUSSION**			
Discussion	23a	Provide a general interpretation of the results in the context of other evidence.	Pages 11, 12
	23b	Discuss any limitations of the evidence included in the review.	Page 11
	23c	Discuss any limitations of the review processes used.	Page 11
	23d	Discuss implications of the results for practice, policy, and future research.	Page 12
**OTHER INFORMATION**			
Registration and protocol	24a	Provide registration information for the review, including register name and registration number, or state that the review was not registered.	Pages 5
	24b	Indicate where the review protocol can be accessed, or state that a protocol was not prepared.	Pages 5
	24c	Describe and explain any amendments to information provided at registration or in the protocol.	None made, explained on page 5
Support	25	Describe sources of financial or non-financial support for the review, and the role of the funders or sponsors in the review.	Page 3
Competing interests	26	Declare any competing interests of review authors.	Page 13
Availability of data, code and other materials	27	Report which of the following are publicly available and where they can be found: template data collection forms; data extracted from included studies; data used for all analyses; analytic code; any other materials used in the review.	Page 14

From: Page MJ, McKenzie JE, Bossuyt PM, Boutron I, Hoffmann TC, Mulrow CD, et al. The PRISMA 2020 statement: an updated guideline for reporting systematic reviews. BMJ 2021;372:n71. doi: 10.1136/bmj.n71.
This work is licensed under CC BY 4.0. To view a copy of this license, visit https://creativecommons.org/licenses/by/4.0/

Supplementary Table 1. Association between any type of ACE and any type of disease

Factor	N cases	Class	OR	CI	p_value	95% PI (PI_eOR)	I2	egger_p	ESB_p	LS 95% OR (largest_CI_eOR)
ACE X any_disease	412760	II	1.58	[1.43, 1.747]	3.12e-19	[0.777, 3.213]	95.496	9.58e-06	0.0	[1.331, 1.578]

Number of studies; N: number of participants; OR: Odds Ratio; ES: Effect Sizes; CI: Confidence Interval; PI: Prediction Interval; I2: statistic denoting between-study heterogeneity; egger: Egger's test; ESB: Excess of Significant Bias; LS: Effect size of study with largest number of participants.

Supplementary Table 2. Association between specific types of ACE and any type of disease

Factor	N cases	Class	OR	CI	p_value	95% PI (PI_eOR)	I2	egger_p	ESB_p	LS 95% OR (largest_CI_eOR)
abuse X any_disease	289,697	II	1.609	[1.498, 1.728]	4.42e-39	[0.824, 3.141]	88.024	3.88e-06	5.55e-16	[3.944, 12.765]
household_dysfunction X any_disease	90,693	III	1.306	[1.164, 1.466]	5.87e-06	[0.807, 2.113]	80.68	5.16e-03	4.57e-05	[1.046, 1.201]
neglect X any_disease	13,245	IV	1.664	[1.157, 2.392]	6.01e-03	[0.486, 5.692]	80.013	4.44e-02	1.05e-04	[0.688, 2.77]
bullying X any_disease	8,487	II	2.037	[1.587, 2.616]	2.36e-08	[0.886, 4.683]	77.964	3.00e-01	4.97e-01	[1.202, 2.401]
physical_abuse X any_disease	225,479	II	1.586	[1.346, 1.869]	3.77e-08	[0.512, 4.914]	97.467	3.01e-01	1.21e-01	[3.314, 12.47]
sexual_abuse X any_disease	215,997	II	1.578	[1.464, 1.701]	1.43e-32	[0.916, 2.718]	75.193	3.30e-06	1.39e-12	[3.914, 15.668]
emotional_abuse X any_disease	21,955	III	1.732	[1.318, 2.276]	8.04e-05	[0.465, 6.451]	91.546	1.80e-01	1.55e-01	[1.345, 1.512]
witness_violence X any_disease	14,333	III	1.608	[1.226, 2.109]	5.93e-04	[0.649, 3.982]	80.572	7.34e-02	2.11e-03	[1.02, 1.39]
family_separation X any_disease	11	IV	5.1	[1.68, 15.481]	4.03e-03	< 3 studies	only 1 study	< 3 studies	NA	[1.68, 15.481]
divorce/separation/death X any_disease	60,181	IV	1.362	[1.126, 1.648]	1.46e-03	[0.739, 2.509]	77.644	1.21e-01	1.38e-04	[1.046, 1.201]
incarcarated_family_member X any_disease	14,538	IV	1.242	[1.006, 1.532]	4.37e-02	[0.6, 2.571]	80.364	1.89e-01	3.87e-01	[0.874, 1.085]
financial_difficulties X any_disease	3,454	ns	1.353	[0.917, 1.997]	1.28e-01	[0.011, 167.14]	88.993	9.84e-01	3.99e-01	[1.038, 1.393]
family_mental_illness X any_disease	1,415	ns	1.291	[0.811, 2.058]	2.82e-01	[0.008, 198.302]	59.44	7.33e-01	3.42e-01	[0.874, 1.371]
family_dysfunction X any_disease	10,724	ns	1.167	[0.983, 1.385]	7.79e-02	< 3 studies	0	< 3 studies	NA	[0.864, 1.457]
substance_use_family X any_disease	14,471	ns	1.141	[0.998, 1.304]	5.34e-02	[0.779, 1.671]	0	2.81e-01	4.93e-01	[1.068, 1.398]

Number of studies; N: number of participants; OR: Odds Ratio; ES: Effect Sizes; CI: Confidence Interval; PI: Prediction Interval; I2: statistic denoting between-study heterogeneity; egger: Egger's test; ESB: Excess of Significant Bias; LS: Effect size of study with largest number of participants.

Supplementary Table 3. Association between any type of ACE and specific types of disease

Factor	N cases	Class	OR	CI	p_value	95% PI (PI_eOR)	I2	egger_p	ESB_p	LS 95% OR (largest_CI_eOR)
ACE X headache	15,898	II	1.909	[1.602, 2.276]	5.45e-13	[0.922, 3.954]	92.098	7.72e-01	4.57e-01	[1.202, 2.401]
ACE X ibs	11,517	II	1.795	[1.449, 2.224]	8.79e-08	[0.711, 4.529]	85.05	3.50e-02	5.69e-03	[1.26, 1.389]
ACE X diabetes	36,350	II	1.667	[1.411, 1.968]	1.73e-09	[0.821, 3.384]	73.714	7.76e-02	9.36e-04	[3.944, 12.765]
ACE X cardiovascular	99,305	II	1.455	[1.272, 1.664]	4.43e-08	[0.719, 2.944]	81.852	9.17e-02	8.95e-02	[1.154, 1.302]
ACE X obesity	145,430	III	1.477	[1.353, 1.612]	2.95e-18	[0.716, 3.048]	97.225	8.48e-01	9.89e-04	[0.874, 1.085]
ACE X respiratory	41,861	ns	1.295	[0.99, 1.693]	5.89e-02	[0.364, 4.606]	96.704	4.78e-01	7.47e-05	[0.958, 1.507]
ACE X somatic_pain	270	IV	2.886	[1.297, 6.42]	9.38e-03	[0.106, 78.435]	71.937	9.27e-01	6.24e-01	[2.984, 9.382]
ACE X pelvic/genital_pain	853	IV	2.186	[1.359, 3.518]	1.26e-03	[0.557, 8.577]	72.887	2.92e-03	4.61e-03	[0.78, 1.23]
ACE X multiple_sclerosis	9,051	IV	1.816	[1.162, 2.838]	8.78e-03	[0.388, 8.504]	88.233	3.25e-02	1.61e-03	[1.046, 1.201]
ACE X dysmenorreia	8,811	IV	1.328	[1.068, 1.653]	1.09e-02	[0.735, 2.401]	53.453	6.48e-01	4.72e-01	[1.209, 1.878]
ACE X uti	1,541	III	1.644	[1.33, 2.032]	4.19e-06	< 3 studies	only 1 study	< 3 studies	NA	[1.33, 2.032]
ACE X hiv	510	IV	2.148	[1.367, 3.377]	9.18e-04	[0.312, 14.779]	81.398	1.84e-01	8.22e-05	[1.033, 1.609]
ACE X cancer	25,009	IV	1.749	[1.191, 2.569]	4.38e-03	[0.4, 7.652]	81.787	2.25e-02	3.20e-01	[1.126, 1.442]
ACE X female_genital_disease	11,779	III	1.115	[1.063, 1.169]	7.93e-06	[1.004, 1.238]	54.203	1.31e-01	1.55e-01	[1.052, 1.163]
ACE X other	6,714	IV	1.809	[1.082, 3.022]	2.37e-02	[0.234, 14]	81.188	8.31e-01	5.21e-01	[0.917, 1.571]

Number of studies; N: number of participants; OR: Odds Ratio; ES: Effect Sizes; CI: Confidence Interval; PI: Prediction Interval; I2: statistic denoting between-study heterogeneity; egger: Egger's test; ESB: Excess of Significant Bias; LS: Effect size of study with largest number of participants.

Supplementary Table 4. Association between any type of ACE and types of disease according to ICD-11 category

Factor	N cases	Class	OR	CI	p_value	95% PI (PI_eOR)	I2	egger_p	ESB_p	LS 95% OR (largest_CI_eOR)
ACE X nervous_system	24,949	II	1.842	[1.558, 2.178]	9.18e-13	[0.816, 4.160]	94.384	1.61e-01	7.67e-03	[1.046, 1.201]
ACE X digestive_system	11,517	II	1.795	[1.449, 2.224]	8.79e-08	[0.711, 4.529]	85.050	3.50e-02	5.69e-03	[1.260, 1.389]
ACE X endocrine_nutritional_metabolic	192,688	II	1.512	[1.398, 1.635]	4.24e-25	[0.741, 3.085]	96.552	6.08e-01	3.57e-07	[3.944, 12.765]
ACE X circulatory_system	99,305	II	1.455	[1.272, 1.664]	4.43e-08	[0.719, 2.944]	81.852	9.17e-02	8.95e-02	[1.154, 1.302]
ACE X genitourinary_system	21,443	III	1.474	[1.228, 1.768]	2.99e-05	[0.766, 2.834]	70.220	4.25e-03	2.02e-04	[1.052, 1.163]
ACE X urinary_system	1,541	III	1.644	[1.330, 2.032]	4.19e-06	< 3 studies	only 1 study	< 3 studies	NA	[1.330, 2.032]
ACE X general_symptoms_pain	270	IV	2.886	[1.297, 6.420]	9.38e-03	[0.106, 78.435]	71.937	9.27e-01	6.24e-01	[2.984, 9.382]
ACE X infectious	510	IV	2.148	[1.367, 3.377]	9.18e-04	[0.312, 14.779]	81.398	1.84e-01	8.22e-05	[1.033, 1.609]
ACE X neoplasms	25,009	IV	1.749	[1.191, 2.569]	4.38e-03	[0.400, 7.652]	81.787	2.25e-02	3.20e-01	[1.126, 1.442]
ACE X other	6,714	IV	1.809	[1.082, 3.022]	2.37e-02	[0.234, 14.000]	81.188	8.31e-01	5.21e-01	[0.917, 1.571]
ACE X respiratory_system	41,861	ns	1.295	[0.990, 1.693]	5.89e-02	[0.364, 4.606]	96.704	4.78e-01	7.47e-05	[0.958, 1.507]

Number of studies; N: number of participants; OR: Odds Ratio; ES: Effect Sizes; CI: Confidence Interval; PI: Prediction Interval; I2: statistic denoting between-study heterogeneity; egger: Egger's test; ESB: Excess of Significant Bias; LS: Effect size of study with largest number of participants.

Supplementary Table 5. Association between specific types of ACE and specific types of disease

Factor	N cases	Class	OR	CI	p_value	95% PI (PI_eOR)	I2	egger_p	ESB_p	LS 95% OR (largest_CI_eOR)
bullying X headache	7,923	II	2.044	[1.554, 2.688]	3.17e-07	[0.807, 5.174]	82.223	2.37e-01	3.17e-01	[1.202, 2.401]
divorce/separation/death X obesity	42,577	II	1.576	[1.356, 1.833]	3.33e-09	[0.593, 4.191]	0	2.52e-01	5.24e-01	[1.178, 2.149]
emotional_abuse X headache	2,094	II	1.573	[1.358, 1.823]	1.64e-09	< 3 studies	20.545	< 3 studies	NA	[1.475, 1.793]

sexual_abuse X obesity	13,550	II	1.449	[1.281, 1.639]	3.47e-09	[0.961, 2.185]	65.907	4.22e-02	6.95e-08	[1.099, 1.269]
sexual_abuse X diabetes	29,328	III	1.764	[1.310, 2.375]	1.87e-04	[0.638, 4.878]	70.894	2.93e-01	1.54e-01	[3.914, 15.668]
sexual_abuse X ibs	2,954	III	1.693	[1.357, 2.112]	3.05e-06	[0.869, 3.297]	58.968	1.26e-01	6.30e-04	[1.088, 2.569]
sexual_abuse X headache	2,983	III	1.668	[1.344, 2.071]	3.54e-06	[0.733, 3.797]	58.475	6.70e-01	3.75e-01	[1.196, 1.625]
financial_difficulties X obesity (RR)	1,551	III	1.607	[1.323, 1.952]	1.71e-06	< 3 studies	only 1 study	< 3 studies	NA	[1.323, 1.952]
emotional_abuse X cancer (RR)	24	IV	6.256	[3.820, 10.245]	3.18e-13	< 3 studies	only 1 study	< 3 studies	NA	[3.820, 10.245]
family_separation X obesity	11	IV	5.100	[1.680, 15.481]	4.03e-03	< 3 studies	only 1 study	< 3 studies	NA	[1.680, 15.481]
sexual_abuse X pelvic/genital_pain	802	IV	2.317	[1.311, 4.094]	3.81e-03	[0.446, 12.029]	75.036	6.80e-03	4.92e-02	[0.780, 1.230]
physical_abuse X multiple_sclerosis	550	ns	4.529	[0.335, 61.306]	2.56e-01	< 3 studies	91.643	< 3 studies	NA	[0.832, 2.061]
bullying X diabetes	487	ns	2.909	[0.661, 12.798]	1.58e-01	< 3 studies	only 1 study	< 3 studies	NA	[0.661, 12.798]
emotional_abuse X multiple_sclerosis	534	ns	2.708	[0.730, 10.045]	1.36e-01	< 3 studies	95.949	< 3 studies	NA	[1.031, 1.900]

Number of studies; N: number of participants; OR: Odds Ratio; ES: Effect Sizes; CI: Confidence Interval; PI: Prediction Interval; I2: statistic denoting between-study heterogeneity; egger: Egger's test; ESB: Excess of Significant Bias; LS: Effect size of study with largest number of participants.

Supplementary Table 6. Association between specific types of ACE and types of disease according to ICD-11 category

Factor	N cases	Class	OR	CI	p_value	95% PI (PI_eOR)	I2	egger_p	ESB_p	LS 95% OR (largest_CI_eOR)
bullying X nervous_system	7,923	II	2.044	[1.554, 2.688]	3.17e-07	[0.807, 5.174]	82.223	2.37e-01	3.17e-01	[1.202, 2.401]
sexual_abuse X endocrine_nutritional_metabolic	42,527	II	1.554	[1.378, 1.752]	6.74e-13	[0.929, 2.600]	72.928	5.80e-02	1.75e-04	[3.914, 15.668]
physical_abuse X endocrine_nutritional_metabolic	31,636	II	1.394	[1.225, 1.586]	4.70e-07	[0.836, 2.323]	78.083	4.52e-01	6.13e-02	[3.314, 12.470]
sexual_abuse X nervous_system	3,651	III	1.694	[1.429, 2.007]	1.24e-09	[1.094, 2.622]	50.446	3.65e-01	3.45e-01	[0.942, 2.046]
sexual_abuse X digestive_system	2,954	III	1.693	[1.357, 2.112]	3.05e-06	[0.869, 3.297]	58.968	1.26e-01	6.30e-04	[1.088, 2.569]

sexual_abuse X urinary_system	1,541	III	1.644	[1.330, 2.032]	4.19e-06	< 3 studies	only 1 study	< 3 studies	NA	[1.330, 2.032]
financial_difficulties X endocrine_nutritional_metabolic (RR)	1,551	III	1.607	[1.323, 1.952]	1.71e-06	< 3 studies	only 1 study	< 3 studies	NA	[1.323, 1.952]
emotional_abuse X neoplasms (RR)	24	IV	6.256	[3.820, 10.245]	3.18e-13	< 3 studies	only 1 study	< 3 studies	NA	[3.820, 10.245]
family_separation X endocrine_nutritional_metabolic	11	IV	5.100	[1.680, 15.481]	4.03e-03	< 3 studies	only 1 study	< 3 studies	NA	[1.680, 15.481]
physical_abuse X nervous_system	6,440	IV	2.685	[1.141, 6.322]	2.37e-02	[0.122, 59.168]	99.421	7.18e-01	8.49e-01	[0.832, 2.061]
sexual_abuse X infectious	510	IV	2.148	[1.367, 3.377]	9.18e-04	[0.312, 14.779]	81.398	1.84e-01	8.22e-05	[1.033, 1.609]
sexual_abuse X respiratory_system	1,739	IV	1.818	[1.149, 2.876]	1.06e-02	< 3 studies	89.905	< 3 studies	NA	[1.201, 1.739]
physical_abuse X circulatory_system	112,686	IV	1.422	[1.150, 1.757]	1.13e-03	[0.672, 3.009]	81.021	4.76e-01	4.60e-02	[1.229, 1.359]
physical_abuse X genitourinary_system	11,779	IV	1.071	[1.007, 1.138]	2.82e-02	[0.872, 1.315]	54.118	6.52e-01	6.79e-01	[1.053, 1.165]
bullying X endocrine_nutritional_metabolic	487	ns	2.909	[0.661, 12.798]	1.58e-01	< 3 studies	only 1 study	< 3 studies	NA	[0.661, 12.798]
physical_abuse X neoplasms	12,841	ns	2.690	[0.809, 8.948]	1.07e-01	[0.009, 819.903]	92.810	3.10e-01	9.30e-03	[1.109, 1.549]
family_mental_illness X endocrine_nutritional_metabolic	487	ns	0.904	[0.390, 2.098]	8.15e-01	< 3 studies	only 1 study	< 3 studies	NA	[0.390, 2.098]

Number of studies; N: number of participants; OR: Odds Ratio; ES: Effect Sizes; CI: Confidence Interval; PI: Prediction Interval; I2: statistic denoting between-study heterogeneity; egger: Egger's test; ESB: Excess of Significant Bias; LS: Effect size of study with largest number of participants.

Supplementary Table 7. List of meta-analyses and systematic reviews included in the global analysis of the association between any adverse childhood experience with any disease.

Factor	Meta-reviews pulled	Number of meta-reviews pulled
ACE X any_disease	afari 2014; amiri 2024; berndt 2024; chen 2023; danese 2014; devyust 2021; duan 2021; elsenburg 2017; gini 2014; hassam 2020; hauser 2011; hemmingsson 2014; holman 2016; hu 2021; huang 2015; jacquet 2022; jakubowski 2021; jianyi liu 2024; joshee 2022; junaid 2023; kaleycheva 2021; lee oh 2018; lenover moya 2025; lloyd 2012; lopes 2020; lopez 2020; mikio moriya 2022; mousauaoi 2022; paras 2009; polick 2022; provencher 2010; rehan 2023; schroeder 2021; selai 2003; souma 2023; zhang 2022; zhang 2023; zhou 2024	38

Supplementary Table 8. List of meta-analyses and systematic reviews included in the analysis of associations between any adverse childhood experience with diseases grouped according to the International Classification of Diseases 11th Revision.

Factor	Meta-reviews pulled	Number of meta-reviews pulled
ACE X endocrine_nutritional_metabolic	amiri 2024; danese 2014; devyust 2021; elsenburg 2017; hemmingsson 2014; huang 2015; jakubowski 2021; kaleycheva 2021; lee oh 2018; mikio moriya 2022; paras 2009; provencher 2010; schroeder 2021; zhang 2022; zhang 2023; zhou 2024	16
ACE X genitourinary_system	berndt 2024; hassam 2020; mousauaoi 2022; paras 2009	4
ACE X circulatory_system	chen 2023; jacquet 2022; jakubowski 2021; souma 2023	4
ACE X nervous_system	gini 2014; jianyi liu 2024; polick 2022; rehan 2023	4
ACE X digestive_system	joshee 2022; junaid 2023; lenover moya 2025	3
ACE X respiratory_system	duan 2021; lee oh 2018; lopez 2020; provencher 2010	4
ACE X neoplasms	holman 2016; hu 2021; lopes 2020	3

ACE X infectious	lloyd 2012	1
ACE X general_symptoms_pain	afari 2014; hauser 2011	2
ACE X other	lee oh 2018; paras 2009; provencher 2010	3
ACE X urinary_system	selai 2003	1

Supplementary Table 9. List of meta-analyses and systematic reviews included in the analysis of associations between any adverse childhood experience with specific diseases.

Factor	Meta-reviews pulled	Number of meta-reviews pulled
ACE X obesity	amiri 2024; danese 2014; elsenburg 2017; hemmingsson 2014; lee oh 2018; mikio moriya 2022; paras 2009; provencher 2010; schroeder 2021; zhou 2024	10
ACE X female_genital_disease	berndt 2024	1
ACE X cardiovascular	chen 2023; jacquet 2022; jakubowski 2021; souma 2023	4
ACE X headache	gini 2014; jianyi liu 2024	2
ACE X ibs	joshee 2022; junaid 2023; lenover moya 2025	3
ACE X multiple_sclerosis	polick 2022; rehan 2023	2
ACE X respiratory	duan 2021; lee oh 2018; lopez 2020; provencher 2010	4
ACE X diabetes	devyust 2021; huang 2015; jakubowski 2021; kaleycheva 2021; zhang 2022; zhang 2023	6
ACE X cancer	holman 2016; hu 2021; lopes 2020	3
ACE X dysmenorreia	mousauaoi 2022	1
ACE X hiv	lloyd 2012	1
ACE X somatic_pain	afari 2014; hauser 2011	2
ACE X other	lee oh 2018; paras 2009; provencher 2010	3
ACE X pelvic/genital_pain	hassam 2020; mousauaoi 2022; paras 2009	3
ACE X uti	selai 2003	1

Supplementary Table 10. List of meta-analyses and systematic reviews included in the analysis of associations between adverse childhood experiences grouped together into abuse, neglect, or household dysfunction, with any diseases.

Factor	Meta-reviews pulled	Number of meta-reviews pulled
abuse X any_disease	afari 2014; berndt 2024; chen 2023; danese 2014; duan 2021; hassam 2020; hauser 2011; hemmingsson 2014; holman 2016; hu 2021; huang 2015; jakubowski 2021; jianyi liu 2024; junaid 2023; kaleycheva 2021; lee oh 2018; lenover moya 2025; lloyd 2012; mousauaoi 2022; paras 2009; polick 2022; rehan 2023; schroeder 2021; selai 2003; zhang 2022; zhou 2024	26
household_dysfunction X any_disease	berndt 2024; devyust 2021; hu 2021; lee oh 2018; mikio moriya 2022; polick 2022; provencher 2010; schroeder 2021; zhang 2022; zhou 2024	10
neglect X any_disease	berndt 2024; danese 2014; hemmingsson 2014; huang 2015; polick 2022; zhang 2022; zhang 2023; zhou 2024	8

Supplementary Table 11. List of meta-analyses and systematic reviews included in the analysis of associations between adverse childhood experiences grouped together into abuse, neglect, or household dysfunction, with specific diseases.

Factor	Meta-reviews pulled	Number of meta-reviews pulled
abuse X female_genital_disease	berndt 2024	1
hosehold_dysfunction X female_genital_disease	berndt 2024	1
neglect X female_genital_disease	berndt 2024	1
abuse X cardiovascular	chen 2023; jakubowski 2021	2
abuse X headache	jianyi liu 2024	1
abuse X ibs	junaid 2023; lenover moya 2025	2
hosehold_dysfunction X obesity	mikio moriya 2022; provencher 2010; schroeder 2021; zhou 2024	4
abuse X multiple_sclerosis	polick 2022; rehan 2023	2
abuse X obesity	danese 2014; hemmingsson 2014; lee oh 2018; paras 2009; schroeder 2021; zhou 2024	6
neglect X obesity	danese 2014; hemmingsson 2014; zhou 2024	3
abuse X cancer	holman 2016; hu 2021	2
hosehold_dysfunction X cancer	hu 2021	1
abuse X dysmenorreia	mousauaoi 2022	1
abuse X diabetes	huang 2015; jakubowski 2021; kaleycheva 2021; zhang 2022	4
abuse X respiratory	duan 2021; lee oh 2018	2
abuse X somatic_pain	afari 2014; hauser 2011	2
abuse X hiv	lloyd 2012	1
hosehold_dysfunction X respiratory	lee oh 2018; provencher 2010	2
hosehold_dysfunction X cardiovascular	provencher 2010	1
abuse X pelvic/genital_pain	hassam 2020; paras 2009	2
neglect X diabetes	huang 2015; zhang 2022; zhang 2023	3
hosehold_dysfunction X diabetes	devyust 2021; zhang 2022	2
abuse X uti	selai 2003	1
hosehold_dysfunction X multiple_sclerosis	polick 2022	1

neglect X multiple_sclerosis	polick 2022	1

Supplementary Table 12. List of meta-analyses and systematic reviews included in the analysis of associations between specific adverse childhood experiences with diseases grouped according to the International Classification of Diseases 11th Revision.

Factor	Meta-reviews pulled	Number of meta-reviews pulled
sexual_abuse X any_disease	afari 2014; berndt 2024; chen 2023; danese 2014; duan 2021; hassam 2020; hauser 2011; hemmingsson 2014; holman 2016; hu 2021; huang 2015; jakubowski 2021; jianyi liu 2024; junaid 2023; kaleycheva 2021; lenover moya 2025; lloyd 2012; mousauaoi 2022; paras 2009; polick 2022; rehan 2023; schroeder 2021; selai 2003; zhang 2022; zhou 2024	25
physical_abuse X any_disease	berndt 2024; chen 2023; duan 2021; hauser 2011; hemmingsson 2014; holman 2016; hu 2021; huang 2015; jianyi liu 2024; kaleycheva 2021; lenover moya 2025; rehan 2023; schroeder 2021; zhang 2022; zhou 2024	15
emotional_abuse X any_disease	berndt 2024; chen 2023; duan 2021; hemmingsson 2014; holman 2016; jianyi liu 2024; kaleycheva 2021; polick 2022; rehan 2023; schroeder 2021; zhang 2022; zhou 2024	12
financial_difficulties X any_disease	hu 2021; mikio moriya 2022	2
divorce/separation/death X any_disease	devyust 2021; hu 2021; polick 2022; schroeder 2021; zhang 2022; zhou 2024	6
family_separation X any_disease	zhou 2024	1
witness_violence X any_disease	duan 2021; hu 2021; schroeder 2021	3

substance_use_family X any_disease	hu 2021; zhang 2022	2
incarcarated_family_member X any_disease	provencher 2010; zhang 2022	2
bullying X any_disease	gini 2014; mousauaoi 2022; zhang 2022	3
family_dysfunction X any_disease	zhang 2022	1
family_mental_illness X any_disease	lee oh 2018; zhang 2022	2

Supplementary Table 13. List of meta-analyses and systematic reviews included in the analysis of associations between specific adverse childhood experiences with any diseases.

Factor	Meta-reviews pulled	Number of meta-reviews pulled
sexual_abuse X genitourinary_system	berndt 2024; hassam 2020; mousauaoi 2022; paras 2009	4
physical_abuse X genitourinary_system	berndt 2024	1
emotional_abuse X genitourinary_system	berndt 2024	1
sexual_abuse X circulatory_system	chen 2023; jakubowski 2021	2
emotional_abuse X circulatory_system	chen 2023	1
physical_abuse X circulatory_system	chen 2023	1
sexual_abuse X nervous_system	jianyi liu 2024; polick 2022; rehan 2023	3
physical_abuse X nervous_system	jianyi liu 2024; rehan 2023	2
emotional_abuse X nervous_system	jianyi liu 2024; polick 2022; rehan 2023	3
sexual_abuse X digestive_system	junaid 2023; lenover moya 2025	2
physical_abuse X digestive_system	lenover moya 2025	1
financial_difficulties X endocrine_nutritional_metabolic	mikio moriya 2022	1

sexual_abuse X endocrine_nutritional_metabolic	danese 2014; hemmingsson 2014; huang 2015; jakubowski 2021; kaleycheva 2021; schroeder 2021; zhang 2022; zhou 2024	8
physical_abuse X endocrine_nutritional_metabolic	hemmingsson 2014; huang 2015; kaleycheva 2021; schroeder 2021; zhang 2022; zhou 2024	6
divorce/separation/death X endocrine_nutritional_metabolic	devyust 2021; schroeder 2021; zhang 2022; zhou 2024	4
emotional_abuse X endocrine_nutritional_metabolic	hemmingsson 2014; kaleycheva 2021; schroeder 2021; zhang 2022; zhou 2024	5
family_separation X endocrine_nutritional_metabolic	zhou 2024	1
sexual_abuse X neoplasms	holman 2016; hu 2021	2
physical_abuse X neoplasms	holman 2016; hu 2021	2
witness_violence X neoplasms	hu 2021	1
substance_use_family X neoplasms	hu 2021	1
divorce/separation/death X neoplasms	hu 2021	1
financial_difficulties X neoplasms	hu 2021	1
sexual_abuse X infectious	lloyd 2012	1
witness_violence X endocrine_nutritional_metabolic	schroeder 2021	1
sexual_abuse X general_symptoms_pain	afari 2014; hauser 2011	2
physical_abuse X general_symptoms_pain	hauser 2011	1
incarcarated_family_member X respiratory_system	provencher 2010	1
physical_abuse X respiratory_system	duan 2021	1
sexual_abuse X respiratory_system	duan 2021	1
witness_violence X respiratory_system	duan 2021	1
incarcarated_family_member X circulatory_system	provencher 2010	1
bullying X nervous_system	gini 2014	1

family_dysfunction X endocrine_nutritional_metabolic	zhang 2022	1
emotional_abuse X neoplasms	holman 2016	1
family_mental_illness X respiratory_system	lee oh 2018	1
sexual_abuse X urinary_system	selai 2003	1
divorce/separation/death X nervous_system	polick 2022	1
substance_use_family X endocrine_nutritional_metabolic	zhang 2022	1
bullying X genitourinary_system	mousauaoi 2022	1
emotional_abuse X respiratory_system	duan 2021	1
bullying X endocrine_nutritional_metabolic	zhang 2022	1
incarcarated_family_member X endocrine_nutritional_metabolic	provencher 2010; zhang 2022	2
family_mental_illness X endocrine_nutritional_metabolic	zhang 2022	1

Supplementary Table 14. List of meta-analyses and systematic reviews included in the analysis of associations between specific adverse childhood experiences with specific diseases.

Factor	Meta-reviews pulled	Number of meta-reviews pulled
sexual_abuse X female_genital_disease	berndt 2024	1
physical_abuse X female_genital_disease	berndt 2024	1
emotional_abuse X female_genital_disease	berndt 2024	1
sexual_abuse X cardiovascular	chen 2023; jakubowski 2021	2
emotional_abuse X cardiovascular	chen 2023	1
physical_abuse X cardiovascular	chen 2023	1
sexual_abuse X headache	jianyi liu 2024	1
physical_abuse X headache	jianyi liu 2024	1
emotional_abuse X headache	jianyi liu 2024	1
sexual_abuse X ibs	junaid 2023; lenover moya 2025	2
physical_abuse X ibs	lenover moya 2025	1
financial_difficulties X obesity	mikio moriya 2022	1
physical_abuse X multiple_sclerosis	rehan 2023	1
emotional_abuse X multiple_sclerosis	polick 2022; rehan 2023	2
sexual_abuse X multiple_sclerosis	polick 2022; rehan 2023	2
sexual_abuse X obesity	danese 2014; hemmingsson 2014; schroeder 2021; zhou 2024	4
physical_abuse X obesity	hemmingsson 2014; schroeder 2021; zhou 2024	3
divorce/separation/death X obesity	schroeder 2021; zhou 2024	2
emotional_abuse X obesity	hemmingsson 2014; schroeder 2021; zhou 2024	3
family_separation X obesity	zhou 2024	1
sexual_abuse X cancer	holman 2016; hu 2021	2
physical_abuse X cancer	holman 2016; hu 2021	2
witness_violence X cancer	hu 2021	1

substance_use_family X cancer	hu 2021	1
divorce/separation/death X cancer	hu 2021	1
sexual_abuse X dysmenorreia	mousauaoi 2022	1
sexual_abuse X diabetes	huang 2015; jakubowski 2021; kaleycheva 2021; zhang 2022	4
financial_difficulties X cancer	hu 2021	1
sexual_abuse X hiv	lloyd 2012	1
witness_violence X obesity	schroeder 2021	1
sexual_abuse X somatic_pain	afari 2014; hauser 2011	2
physical_abuse X somatic_pain	hauser 2011	1
incarcarated_family_member X respiratory	provencher 2010	1
physical_abuse X respiratory	duan 2021	1
sexual_abuse X respiratory	duan 2021	1
witness_violence X respiratory	duan 2021	1
incarcarated_family_member X cardiovascular	provencher 2010	1
sexual_abuse X pelvic/genital_pain	hassam 2020; paras 2009	2
bullying X headache	gini 2014	1
family_dysfunction X diabetes	zhang 2022	1
physical_abuse X diabetes	huang 2015; kaleycheva 2021; zhang 2022	3
emotional_abuse X diabetes	kaleycheva 2021; zhang 2022	2
emotional_abuse X cancer	holman 2016	1
divorce/separation/death X diabetes	devyust 2021; zhang 2022	2
family_mental_illness X respiratory	lee oh 2018	1
sexual_abuse X uti	selai 2003	1
divorce/separation/death X multiple_sclerosis	polick 2022	1
substance_use_family X diabetes	zhang 2022	1
bullying X dysmenorreia	mousauaoi 2022	1
emotional_abuse X respiratory	duan 2021	1
bullying X diabetes	zhang 2022	1
incarcarated_family_member X diabetes	zhang 2022	1

family_mental_illness X diabetes	zhang 2022	1
incarcarated_family_member X obesity	provencher 2010	1

Supplementary Table 15. Results from female-only sample data

Factor	N studies	N cases	N controls	Class	OR	CI	p_value	95% PI (PI_eOR)	I2	egger_p	ESB_p	LS 95% OR (largest_CI_eOR)
ACE X any_disease	75	84809	525907	II	1.58	[1.43, 1.747]	3.12e-19	[0.777, 3.213]	95.496	9.58e-06	0.0	[1.331, 1.578]
abuse X any_disease	60	97070	476536	II	1.564	[1.344, 1.82]	6.98e-09	[0.547, 4.47]	99.195	0.0108	8.63e-12	[1.331, 1.578]
sexual_abuse X any_disease	46	37189	452851	II	1.519	[1.368, 1.687]	5.9e-15	[0.892, 2.589]	71.47	3.10e-05	2.82e-08	[1.159, 1.462]
physical_abuse X any_disease	22	46832	376570	II	1.309	[1.182, 1.449]	2.23e-07	[0.887, 1.933]	86.292	1.61e-01	0.382	[1.359, 1.652]
physical_abuse_and_sexual_abuse X any_disease	1	9823	50792	II	0.121	[0.115, 0.127]	0.0	< 3 studies	only 1 study	< 3 studies	nan	[0.115, 0.127]
bullying X any_disease	1	77	138	ns	1.738	[0.819, 3.692]	0.15	< 3 studies	only 1 study	< 3 studies	nan	[0.819, 3.692]
ACE X diabetes	5	2479	66193	IV	1.757	[1.242, 2.487]	0.00145	[0.642, 4.809]	48.906	3.61e-02	0.00553	[1.247, 1.455]
ACE X headache	4	483	13257	IV	1.484	[1.321, 1.668]	2.94e-11	[1.149, 1.917]	0.0	3.34e-01	0.0523	[1.28, 1.662]
ACE X cardiovascular	6	4347	159603	IV	1.433	[1.008, 2.038]	0.0449	[0.448, 4.591]	81.963	9.18e-01	0.697	[1.331, 1.578]
ACE X ibs	2	207	118	ns	6.479	[0.357, 117.652]	0.206	< 3 studies	86.286	< 3 studies	nan	[0.985, 3.081]
ACE X endocrine_nutritional_metabolic	27	24492	149875	II	1.626	[1.402, 1.885]	1.19e-10	[0.82, 3.223]	85.453	3.33e-02	4.71e-10	[1.247, 1.455]
ACE X nervous_system	7	2396	81037	III	1.513	[1.182, 1.935]	0.000991	[0.738, 3.1]	73.739	1.99e-01	0.00016	[1.104, 2.909]
ACE X circulatory_system	6	4347	159603	IV	1.433	[1.008, 2.038]	0.0449	[0.448, 4.591]	81.963	9.18e-01	0.697	[1.331, 1.578]
ACE X digestive_system	2	207	118	ns	6.479	[0.357, 117.652]	0.206	< 3 studies	86.286	< 3 studies	nan	[0.985, 3.081]
abuse X obesity	19	31112	82194	II	1.618	[1.341, 1.952]	4.96e-07	[0.731, 3.581]	91.819	0.0156	3.9e-12	[1.048, 1.138]
physical_abuse_and_sexual_abuse X female_genital_disease	1	9823	50792	II	0.121	[0.115, 0.127]	0.0	< 3 studies	only 1 study	< 3 studies	nan	[0.115, 0.127]

sexual_abuse X obesity	9	7944	41752	III	1.359	[1.186, 1.559]	1.06e-05	[0.954, 1.938]	54.947	1.64e-02	1.69e-08	[1.099, 1.269]
divorce/separation/death X obesity	1	725	5954	IV	1.611	[1.347, 1.926]	1.75e-07	< 3 studies	only 1 study	< 3 studies	nan	[1.347, 1.926]
emotional_abuse X headache	1	167	620	ns	1.326	[0.942, 1.868]	0.106	< 3 studies	only 1 study	< 3 studies	nan	[0.942, 1.868]
sexual_abuse X endocrine_nutritional_metabolic	14	10068	107702	II	1.441	[1.251, 1.659]	4.05e-07	[0.978, 2.122]	57.303	1.34e-02	9.07e-05	[1.274, 1.522]
physical_abuse_and_sexual_abuse X genitourinary_system	1	9823	50792	II	0.121	[0.115, 0.127]	0.0	< 3 studies	only 1 study	< 3 studies	nan	[0.115, 0.127]
physical_abuse X endocrine_nutritional_metabolic	10	4797	75032	III	1.456	[1.219, 1.741]	3.53e-05	[0.879, 2.413]	78.561	6.38e-01	0.0658	[1.192, 1.425]

Number of studies; N: number of participants; OR: Odds Ratio; ES: Effect Sizes; CI: Confidence Interval; PI: Prediction Interval; I2: statistic denoting between-study heterogeneity; egger: Egger's test; ESB: Excess of Significant Bias; LS: Effect size of study with largest number of participants.

Supplementary Table 16. Results from male-only sample data

Factor	N cases	Class	OR	CI	p_value	95% PI (PI_eOR)	I2	egger_p	ESB_p	LS 95% OR (largest_CI_eOR)
ACE X any_disease	30,570	III	1.417	[1.179, 1.703]	2.05e-04	[0.654, 3.070]	79.607	1.85e-01	4.00e-03	[1.182, 1.344]
abuse X any_disease	29,511	III	1.447	[1.180, 1.775]	3.91e-04	[0.644, 3.252]	78.643	2.47e-01	3.59e-03	[1.182, 1.344]
sexual_abuse X any_disease	21,269	III	1.544	[1.211, 1.968]	4.56e-04	[0.663, 3.593]	78.417	1.91e-01	2.42e-04	[1.089, 1.336]
physical_abuse X any_disease	31,072	IV	1.254	[1.091, 1.442]	1.50e-03	[0.802, 1.962]	67.994	6.82e-01	5.67e-01	[1.155, 1.299]
ACE X any_disease	30,570	III	1.417	[1.179, 1.703]	2.05e-04	[0.654, 3.070]	79.607	1.85e-01	4.00e-03	[1.182, 1.344]
abuse X any_disease	29,511	III	1.447	[1.180, 1.775]	3.91e-04	[0.644, 3.252]	78.643	2.47e-01	3.59e-03	[1.182, 1.344]
sexual_abuse X any_disease	21,269	III	1.544	[1.211, 1.968]	4.56e-04	[0.663, 3.593]	78.417	1.91e-01	2.42e-04	[1.089, 1.336]
physical_abuse X any_disease	31,072	IV	1.254	[1.091, 1.442]	1.50e-03	[0.802, 1.962]	67.994	6.82e-01	5.67e-01	[1.155, 1.299]
abuse X obesity	8,984	ns	1.362	[0.904, 2.050]	1.39e-01	[0.338, 5.479]	85.247	9.23e-01	1.31e-01	[1.856, 3.000]
sexual_abuse X endocrine_nutritional_metabolic	3,540	ns	1.343	[0.802, 2.249]	2.62e-01	[0.135, 13.399]	81.273	4.27e-01	5.05e-01	[1.817, 3.418]
physical_abuse X endocrine_nutritional_metabolic	1,970	ns	1.320	[0.770, 2.264]	3.13e-01	< 3 studies	89.228	< 3 studies	NA	[1.292, 2.421]
sexual_abuse X obesity	3,540	ns	1.343	[0.802, 2.249]	2.62e-01	[0.135, 13.399]	81.273	4.27e-01	5.05e-01	[1.817, 3.418]

Number of studies; N: number of participants; OR: Odds Ratio; ES: Effect Sizes; CI: Confidence Interval; PI: Prediction Interval; I2: statistic denoting between-study heterogeneity; egger: Egger's test; ESB: Excess of Significant Bias; LS: Effect size of study with largest number of participants.

Supplementary Table 17. Associations from studies with both trauma and disease measured prospectively

Factor	N cases	Class	OR	CI	p_value	95% PI (PI_eOR)	I2	egger_p	ESB_p	LS 95% OR (largest_CI_eOR)
ACE X any_disease	24,666	II	1.473	[1.293, 1.678]	5.33e-09	[0.907, 2.392]	89.679	5.66e-02	6.76e-05	[1.052, 1.163]
ACE X endocrine_nutritional_metabolic	6,015	I	1.624	[1.424, 1.852]	4.48e-13	[1.150, 2.295]	46.348	3.40e-01	1.24e-01	[1.189, 2.049]

ACE X nervous_system	3,362	ns	1.322	[0.968, 1.803]	7.88e-02	< 3 studies	37.971	< 3 studies	NA	[0.916, 1.520]
ACE X headache	102	IV	1.650	[1.051, 2.590]	2.97e-02	< 3 studies	only 1 study	< 3 studies	NA	[1.051, 2.590]
ACE X diabetes	888	IV	1.648	[1.215, 2.237]	1.32e-03	< 3 studies	49.073	< 3 studies	NA	[1.039, 1.918]
abuse X any_disease	16,973	III	1.489	[1.184, 1.871]	6.54e-04	[0.679, 3.264]	94.131	2.09e-01	5.34e-02	[1.052, 1.163]
abuse X obesity	263	IV	1.934	[1.313, 2.850]	8.41e-04	< 3 studies	only 1 study	< 3 studies	NA	[1.313, 2.850]
physical_abuse X any_disease	12,289	III	1.102	[1.051, 1.157]	7.15e-05	[0.807, 1.506]	0	9.29e-01	6.18e-01	[1.053, 1.165]
bullying X any_disease	102	IV	1.650	[1.051, 2.590]	2.97e-02	< 3 studies	only 1 study	< 3 studies	NA	[1.051, 2.590]
sexual_abuse X any_disease	12,289	IV	1.180	[1.027, 1.356]	1.93e-02	[0.290, 4.804]	51.949	1.22e-01	1.73e-06	[1.036, 1.179]
bullying X nervous_system	102	IV	1.650	[1.051, 2.590]	2.97e-02	< 3 studies	only 1 study	< 3 studies	NA	[1.051, 2.590]

Number of studies; N: number of participants; OR: Odds Ratio; ES: Effect Sizes; CI: Confidence Interval; PI: Prediction Interval; I2: statistic denoting between-study heterogeneity; egger: Egger's test; ESB: Excess of Significant Bias; LS: Effect size of study with largest number of participants.

Supplementary Table 18. Associations from studies with disease measured prospectively and trauma measured retrospectively

**Factor**	**N cases**	**Class**	**OR**	**CI**	**p_value**	**95% PI (PI_eOR)**	**I2**	**egger_p**	**ESB_p**	**LS 95% OR (largest_CI_eOR)**
ACE X any_disease	95,805	II	1.335	[1.261, 1.414]	5.05e-23	[0.967, 1.843]	77.937	6.79e-03	2.17e-03	[1.260, 1.389]
ACE X endocrine_nutritional_metabolic	31,341	II	1.411	[1.291, 1.542]	2.88e-14	[0.968, 2.057]	76.654	4.45e-04	3.07e-07	[1.247, 1.455]
ACE X cardiovascular	24,859	I	1.189	[1.120, 1.262]	1.37e-08	[1.068, 1.324]	13.142	9.06e-01	5.61e-01	[1.154, 1.302]
ACE X digestive_system	7,947	II	1.296	[1.188, 1.414]	5.45e-09	< 3 studies	27.330	< 3 studies	NA	[1.260, 1.389]
ACE X headache	1,028	II	1.612	[1.382, 1.881]	1.26e-09	< 3 studies	0	< 3 studies	NA	[1.364, 1.893]
ACE X ibs	7,947	II	1.296	[1.188, 1.414]	5.45e-09	< 3 studies	27.330	< 3 studies	NA	[1.260, 1.389]
ACE X diabetes	4,141	III	1.392	[1.144, 1.695]	9.75e-04	[0.616, 3.148]	60.630	7.60e-01	2.38e-01	[1.247, 1.455]

ACE X nervous_system	4,288	IV	1.449	[1.159, 1.812]	1.15e-03	[0.142, 14.831]	53.174	8.39e-01	5.49e-01	[0.916, 1.520]
abuse X any_disease	37,285	II	1.391	[1.251, 1.546]	1.01e-09	[0.937, 2.065]	82.889	8.72e-02	4.64e-02	[1.052, 1.163]
abuse X obesity	7,483	III	1.393	[1.192, 1.628]	3.02e-05	[0.989, 1.962]	47.320	6.47e-01	3.27e-01	[0.302, 1.609]
sexual_abuse X any_disease	31,302	II	1.235	[1.147, 1.331]	2.44e-08	[1.018, 1.500]	56.621	2.86e-01	3.59e-02	[1.036, 1.179]
physical_abuse X any_disease	18,858	III	1.271	[1.111, 1.453]	4.66e-04	[0.844, 1.913]	77.517	1.57e-01	8.85e-02	[1.053, 1.165]
sexual_abuse X endocrine_nutritional_metabolic	9,060	III	1.284	[1.155, 1.428]	3.98e-06	[0.983, 1.678]	59.513	7.47e-01	4.75e-01	[1.274, 1.522]
physical_abuse X endocrine_nutritional_metabolic	5,130	III	1.406	[1.200, 1.647]	2.46e-05	[0.956, 2.068]	45.049	1.91e-01	3.90e-02	[1.192, 1.425]
sexual_abuse X obesity	6,993	III	1.211	[1.106, 1.325]	3.37e-05	[0.935, 1.568]	16.031	9.75e-01	3.62e-01	[1.099, 1.269]
bullying X any_disease	102	IV	1.650	[1.051, 2.590]	2.97e-02	< 3 studies	only 1 study	< 3 studies	NA	[1.051, 2.590]
bullying X nervous_system	102	IV	1.650	[1.051, 2.590]	2.97e-02	< 3 studies	only 1 study	< 3 studies	NA	[1.051, 2.590]
bullying X headache	102	IV	1.650	[1.051, 2.590]	2.97e-02	< 3 studies	only 1 study	< 3 studies	NA	[1.051, 2.590]
divorce/separation/death X obesity	57	ns	0.976	[0.445, 2.137]	9.51e-01	< 3 studies	only 1 study	< 3 studies	NA	[0.445, 2.137]

Number of studies; N: number of participants; OR: Odds Ratio; ES: Effect Sizes; CI: Confidence Interval; PI: Prediction Interval; I2: statistic denoting between-study heterogeneity; egger: Egger's test; ESB: Excess of Significant Bias; LS: Effect size of study with largest number of participants.

Supplementary Table 19: Changes in evidence classification after restriction to high-quality reviews (AMSTAR ≥7), according to Ioannidis' criteria. Only associations reported in the main manuscript that changed classification after sensitivity analysis are presented.

ACE - Disease association	Initial evidence class	Sensitivity analysis (AMSTAR ≥7)
Physical abuse and any type of non-mental medical disease	II	III
Witnessing violence and any type of non-mental medical disease	III	IV
Sexual abuse and obesity	II	IV
Emotional abuse and any type of non-mental medical disease	III	II
Any type of ACE and respiratory disease	Non-significant	IV
Any type of ACE and obesity	III	II

Supplementary table 20. AMSTAR scores by item.

**AMSTAR**	1	2	3	4	5	6	7	8	9	10	11	**Total score**
Afari 2014	0	1	1	0	0	1	1	1	1	1	1	8
Amiri 2024	1	0	1	1	0	0	0	0	1	1	1	6
Berndt 2024	1	1	1	0	0	1	1	0	1	0	1	7
Chen 2023	1	1	1	0	0	1	1	0	1	1	1	8
Danese 2014	0	1	1	0	0	1	1	1	1	1	1	8
Devuyst 2021	0	0	1	0	0	1	1	0	0	0	1	4
Duan 2021	1	0	1	0	0	1	1	1	1	1	1	8
Elsenburg 2015	1	1	1	0	0	1	1	1	1	1	1	9
Gini 2014	0	1	1	0	1	1	1	1	1	1	1	9
Hassam 2020	0	1	1	0	0	0	1	1	1	1	1	7

Hauser 2011	0	1	1	0	0	1	1	1	1	1	0	7
Hemmingsson 2014	0	0	1	0	0	1	0	0	1	1	1	5
Holman 2016	0	0	0	0	1	1	0	0	1	0	1	4
Hu 2021	0	1	1	0	0	1	1	0	1	1	1	7
Huang 2015	0	1	1	0	0	1	1	0	1	1	1	7
Jacquet-Smailovic 2022	1	1	1	1	1	1	1	0	1	1	1	10
Jakubowski 2021	1	N/I	1	0	0	1	1	0	1	1	1	7
Jianyi Liu 2024	1	1	1	1	0	1	1	1	1	1	1	10
Junaid_2023	0	1	1	1	0	1	1	1	1	1	0	8
Joshee 2022	0	0	1	1	0	1	1	1	1	1	1	8
Kaleycheva 2021	0	1	1	1	1	1	1	1	1	1	1	10
Lenover Moya_2025	0	1	1	0	0	0	1	1	1	1	1	7
Lloyd 2012	0	0	1	1	1	1	1	0	1	0	1	7
Lopes 2020	0	0	0	0	1	1	1	1	1	0	1	6
Moussaoui 2022	1	0	0	0	0	1	1	1	0	0	1	5
Mikio Moriya_2022	1	1	1	0	0	1	1	1	1	1	0	8
Oh 2018	0	0	1	0	0	1	1	0	0	0	0	3
Paras 2009	0	0	0	0	0	1	1	1	1	1	0	5
Polick 2022	0	0	1	0	0	1	1	1	0	0	1	5
Provencher 2019	0	0	1	1	0	1	1	0	1	0	1	6
Rehan 2023	1	1	1	1	0	1	1	0	0	0	1	7
Schroeder 2021	1	0	1	0	0	1	1	1	0	0	1	6
Selai 2023	1	1	1	0	0	1	1	1	0	0	1	7
Souama 2023	1	0	0	0	0	1	1	1	1	0	1	6
Zhang 2022	0	1	1	0	1	1	1	1	1	1	1	9
Zhou_2024	1	1	1	0	0	1	1	0	1	1	1	8

Key of each item. 1. Was an 'a priori' design provided?; 2. Was there duplicate study selection and data extraction?; 3. Was a comprehensive literature search performed?; 4. Was the status of publication (i.e. grey literature) used as an inclusion criterion?; 5. Was a list of studies (included and excluded) provided?; 6. Were the characteristics of the included studies provided?; 7. Was the scientific quality of the included studies assessed and documented?; 8. Was the scientific quality of the included studies used appropriately in formulating conclusions?; 9. Were the methods used to combine the findings of studies appropriate?; 10. Was the likelihood of publication bias assessed?; 11. Was the conflict of interest included?

Supplementary Figure 1. Association between any type of ACE and any type of physical disease

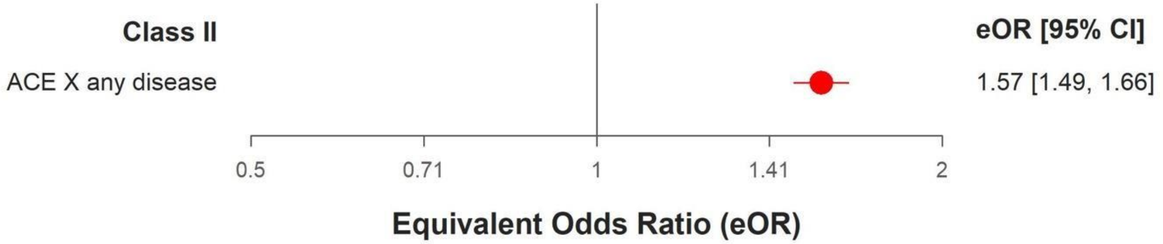

Supplementary Figure 2. Associations between any type of ACE and specific diseases

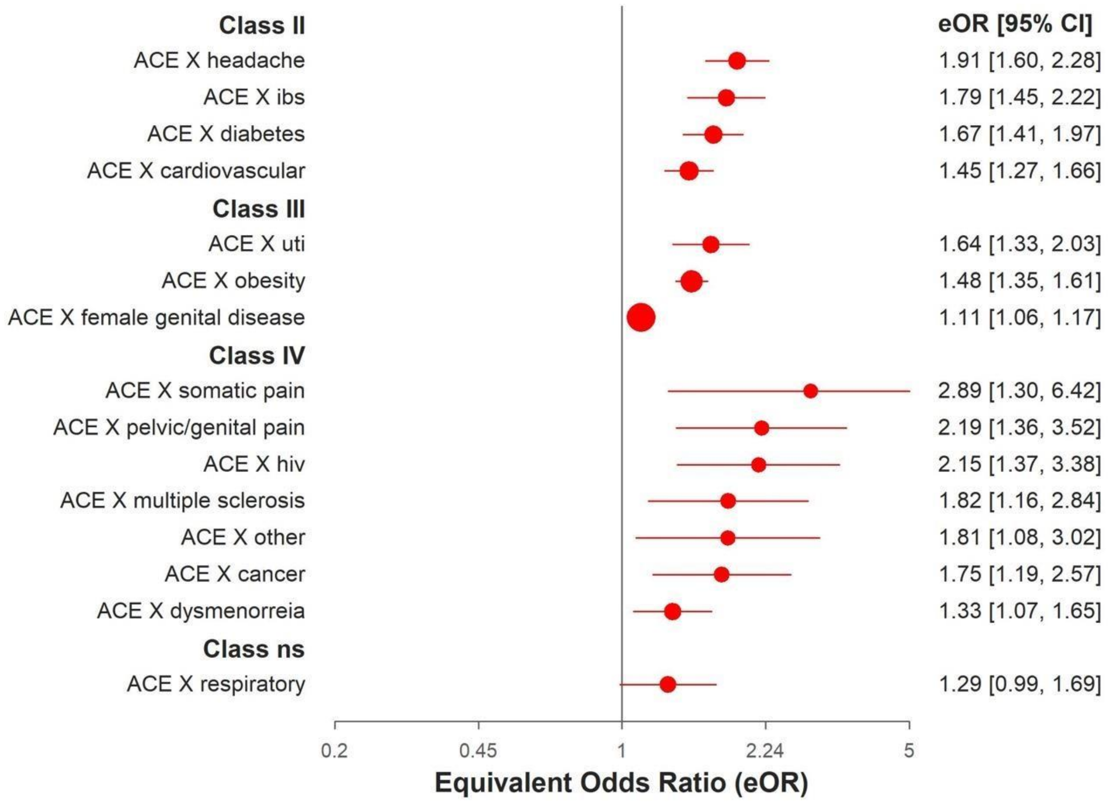

Class II
ACE X headache
ACE X ibs
ACE X diabetes
ACE X cardiovascular
Class III
ACE X uti
ACE X obesity
ACE X female genital disease
Class IV
ACE X somatic pain
ACE X pelvic/genital pain
ACE X hiv
ACE X multiple sclerosis
ACE X other
ACE X cancer
ACE X dysmenorreia
Class ns
ACE X respiratory
eOR [95% CI]
1.91 [1.60, 2.28]
1.79 [1.45, 2.22]
1.67 [1.41, 1.97]
1.45 [1.27, 1.66]
1.64 [1.33, 2.03]
1.48 [1.35, 1.61]
1.11 [1.06, 1.17]
2.89 [1.30, 6.42]
2.19 [1.36, 3.52]
2.15 [1.37, 3.38]
1.82 [1.16, 2.84]
1.81 [1.08, 3.02]
1.75 [1.19, 2.57]
1.33 [1.07, 1.65]
1.29 [0.99, 1.69]
0.2
0.45
1
2.24
5
Equivalent Odds Ratio (eOR)

Supplementary Figure 3. Associations between specific types of ACE and specific types of physical disease

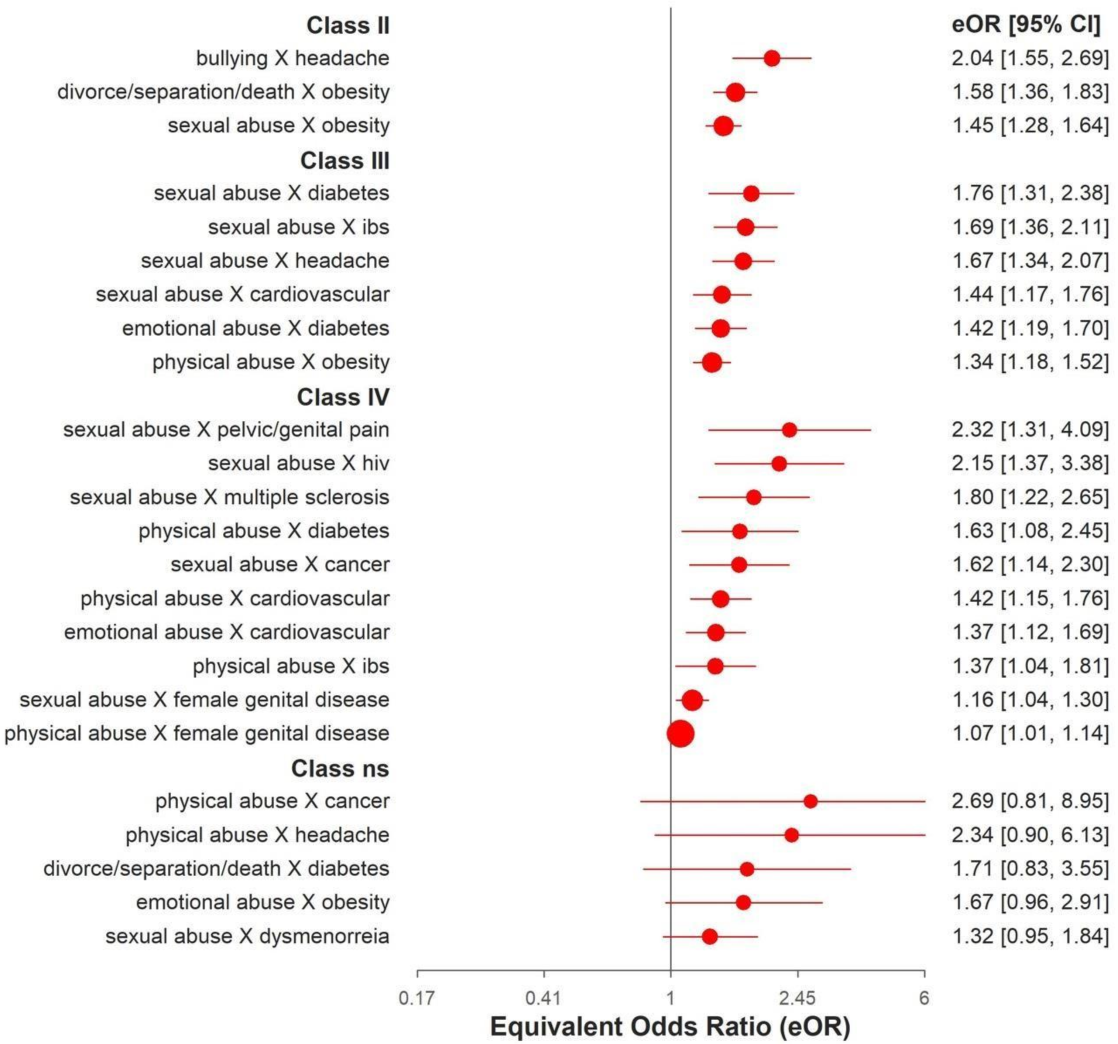
Class II
bullying X headache
divorce/separation/death X obesity
sexual abuse X obesity
Class III
sexual abuse X diabetes
sexual abuse X ibs
sexual abuse X headache
sexual abuse X cardiovascular
emotional abuse X diabetes
physical abuse X obesity
Class IV
sexual abuse X pelvic/genital pain
sexual abuse X hiv
sexual abuse X multiple sclerosis
physical abuse X diabetes
sexual abuse X cancer
physical abuse X cardiovascular
emotional abuse X cardiovascular
physical abuse X ibs
sexual abuse X female genital disease
physical abuse X female genital disease
Class ns
physical abuse X cancer
physical abuse X headache
divorce/separation/death X diabetes
emotional abuse X obesity
sexual abuse X dysmenorreia
eOR [95% CI]
2.04 [1.55, 2.69]
1.58 [1.36, 1.83]
1.45 [1.28, 1.64]
1.76 [1.31, 2.38]
1.69 [1.36, 2.11]
1.67 [1.34, 2.07]
1.44 [1.17, 1.76]
1.42 [1.19, 1.70]
1.34 [1.18, 1.52]
2.32 [1.31, 4.09]
2.15 [1.37, 3.38]
1.80 [1.22, 2.65]
1.63 [1.08, 2.45]
1.62 [1.14, 2.30]
1.42 [1.15, 1.76]
1.37 [1.12, 1.69]
1.37 [1.04, 1.81]
1.16 [1.04, 1.30]
1.07 [1.01, 1.14]
2.69 [0.81, 8.95]
2.34 [0.90, 6.13]
1.71 [0.83, 3.55]
1.67 [0.96, 2.91]
1.32 [0.95, 1.84]
0.17
0.41
1
2.45
6
Equivalent Odds Ratio (eOR)

Supplementary Figure 4. Funnel plot for the association between any type of ACE and any type of disease (Class II)

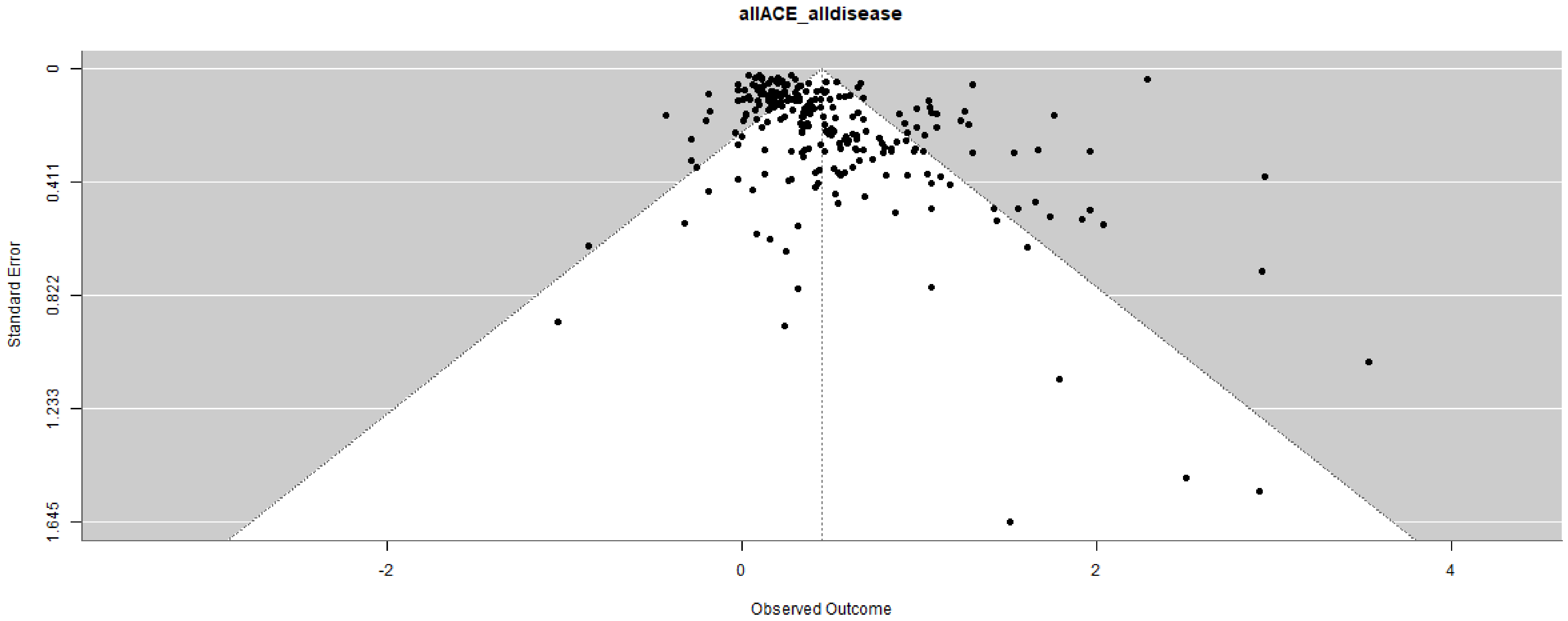

Supplementary Figure 5. Funnel plot for the association between any type of ACE and circulatory system diseases (Class II).

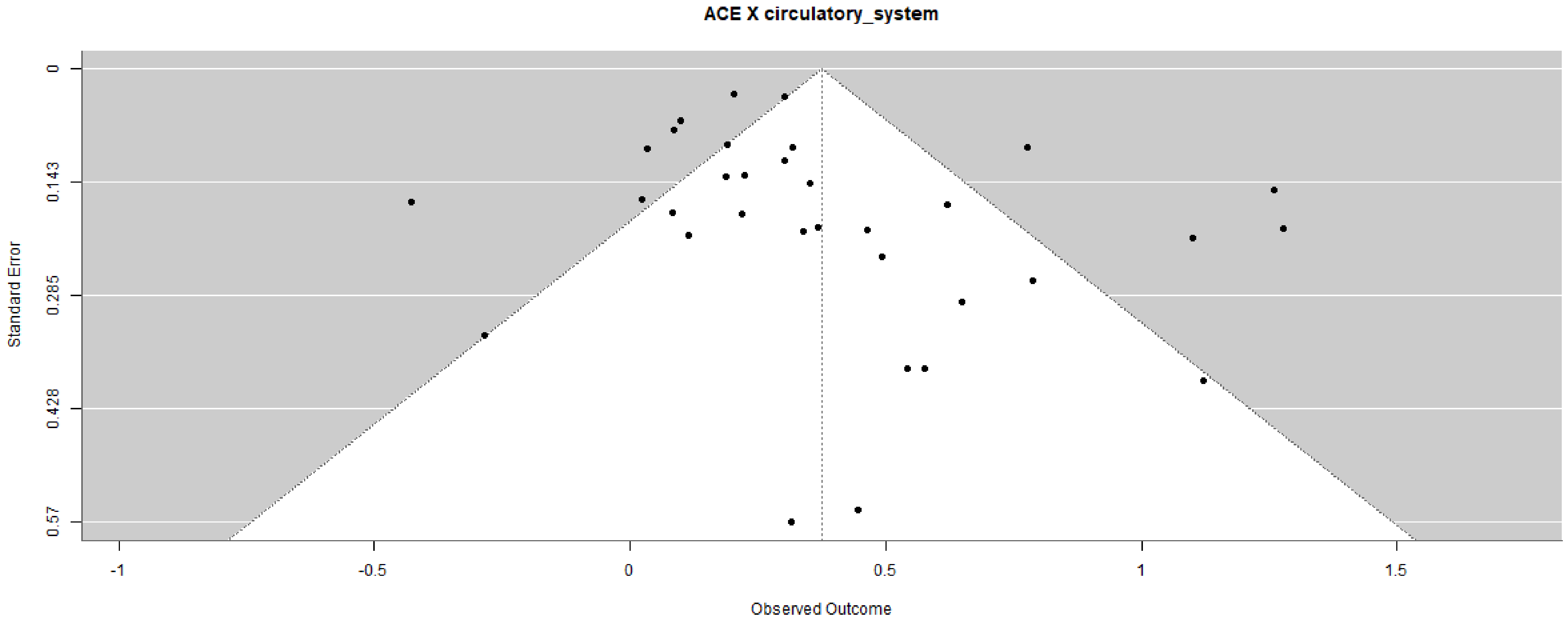

Supplementary Figure 6. Funnel plot for the association between any type of ACE and digestive system diseases (Class II).

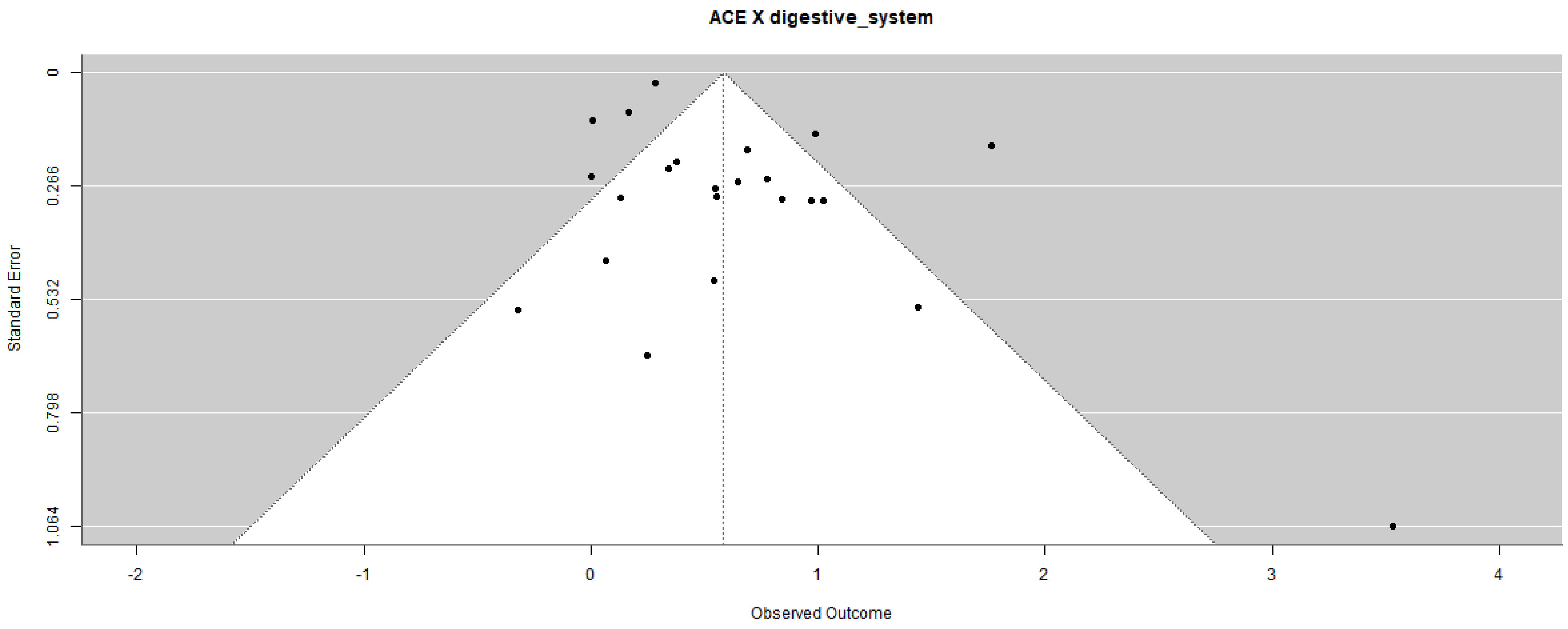

Supplementary Figure 7. Funnel plot for the association between any type of ACE and endocrine/nutritional/metabolic diseases (Class II).

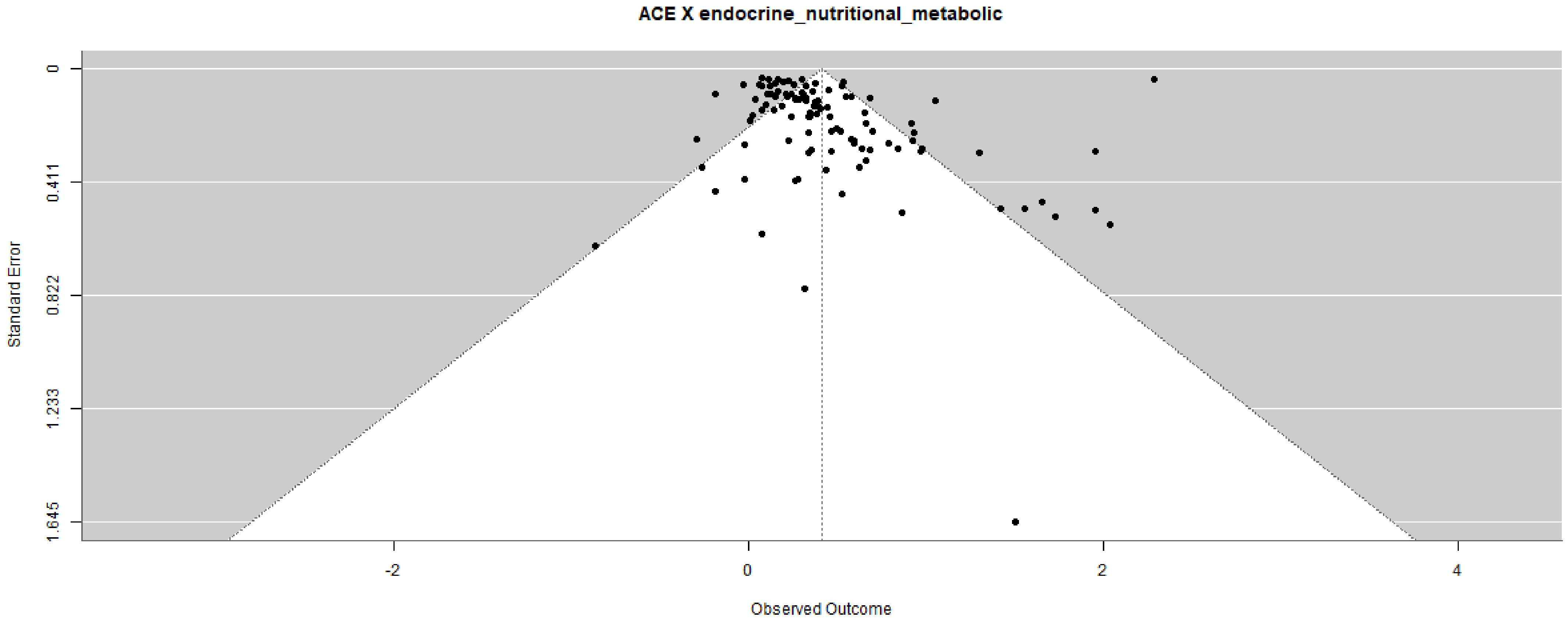

Supplementary Figure 8. Funnel plot for the association between ACE and nervous system diseases (Class II).

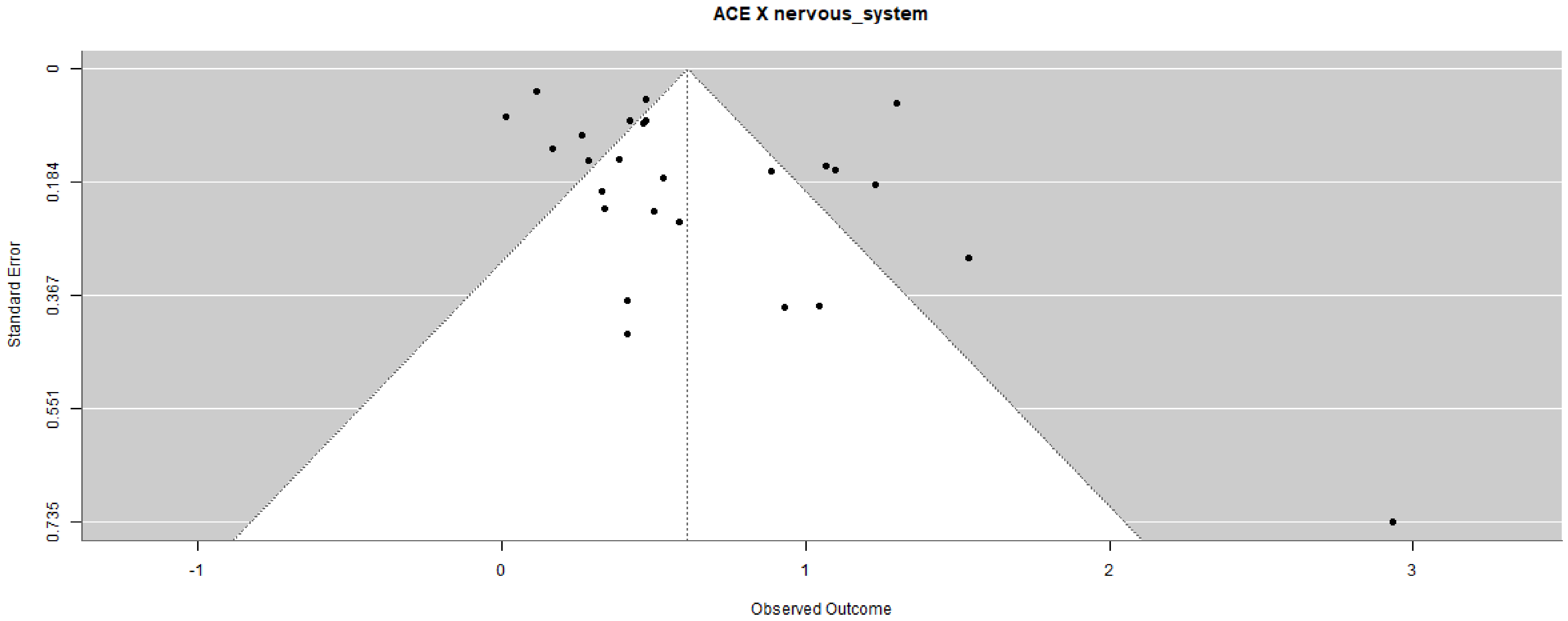

Supplementary Figure 9. Funnel plot for the association between any type of ACE and cardiovascular diseases (Class II).

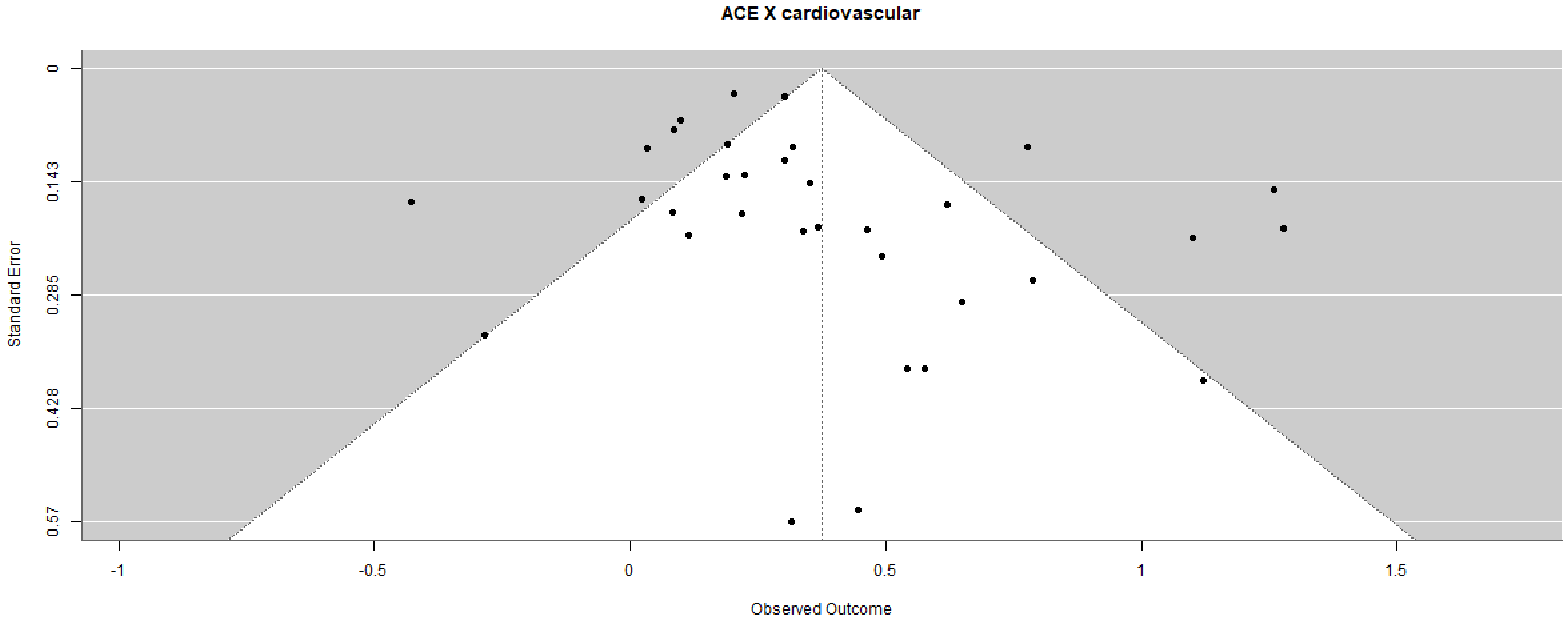

Supplementary Figure 10. Funnel plot for the association between any type of ACE and diabetes (Class II).

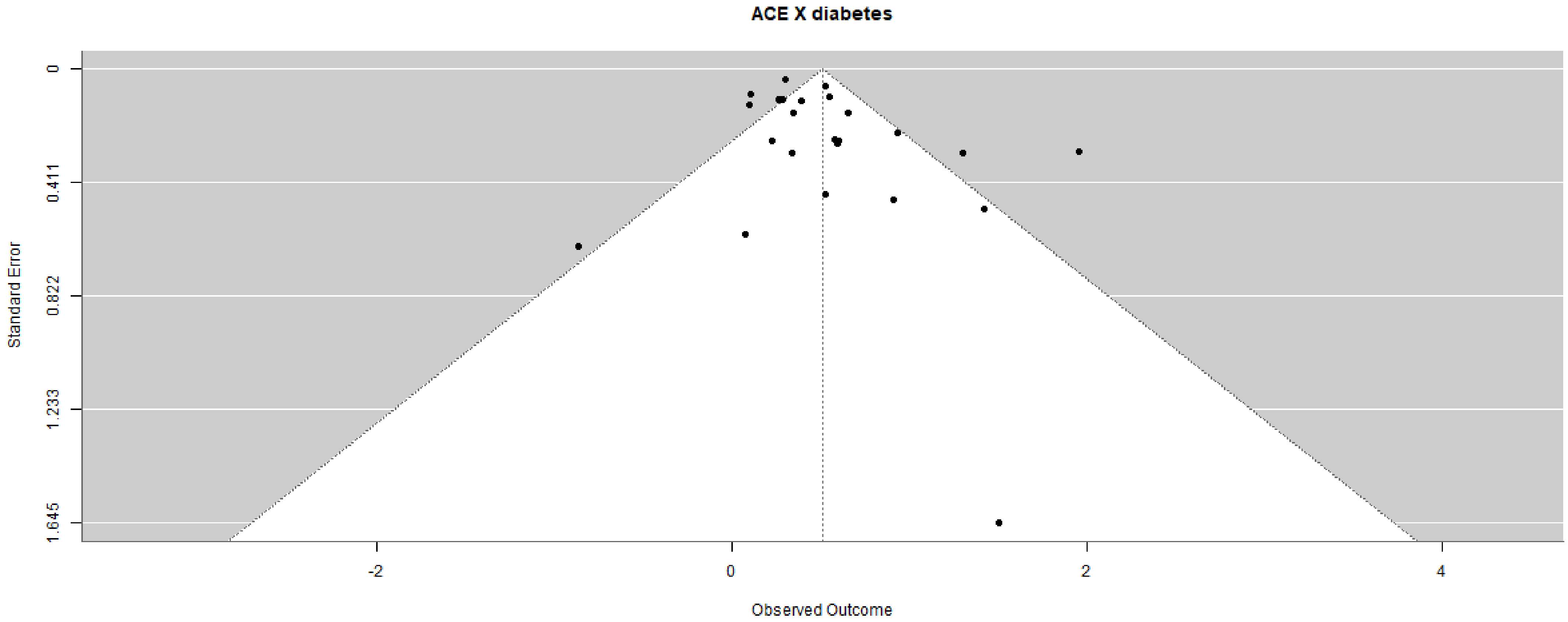

Supplementary Figure 11. Funnel plot for the association between any type of ACE and headache (Class II).

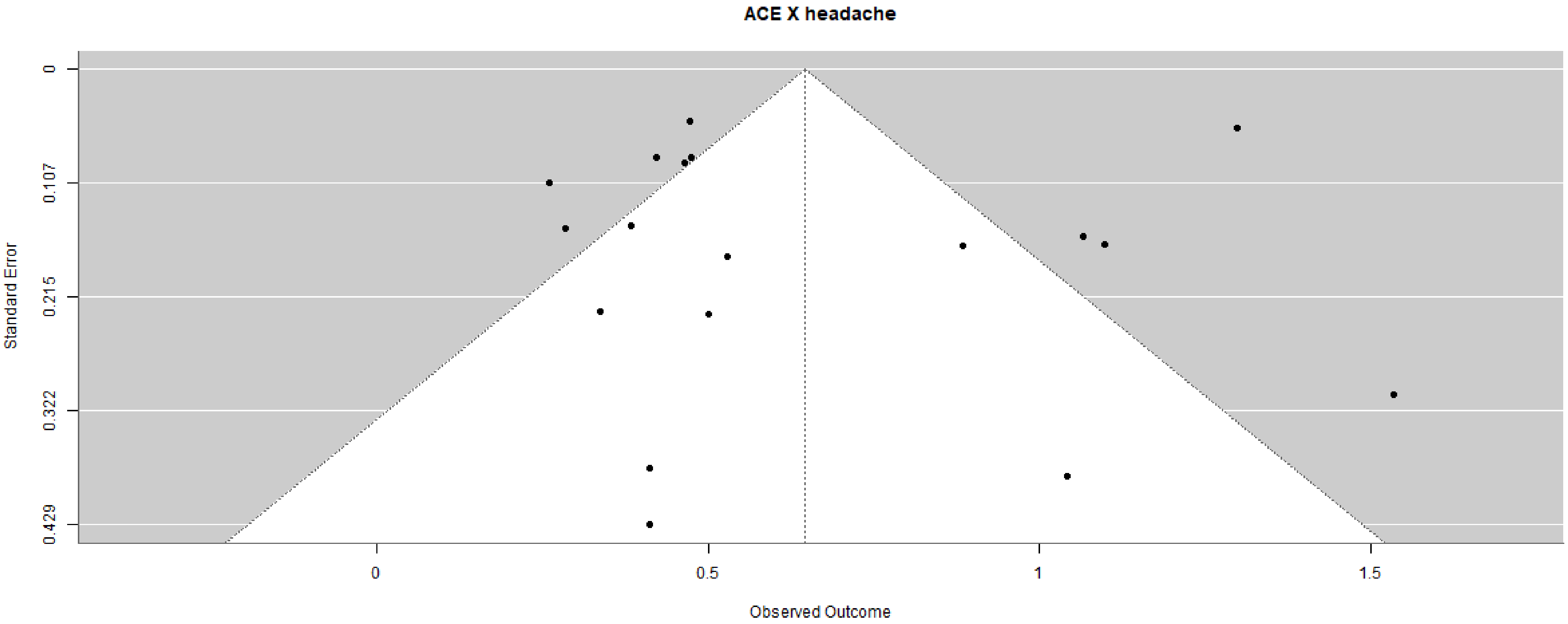

Supplementary Figure 12. Funnel plot for the association between any type of ACE and Irritable Bowel Syndrome (Class II).

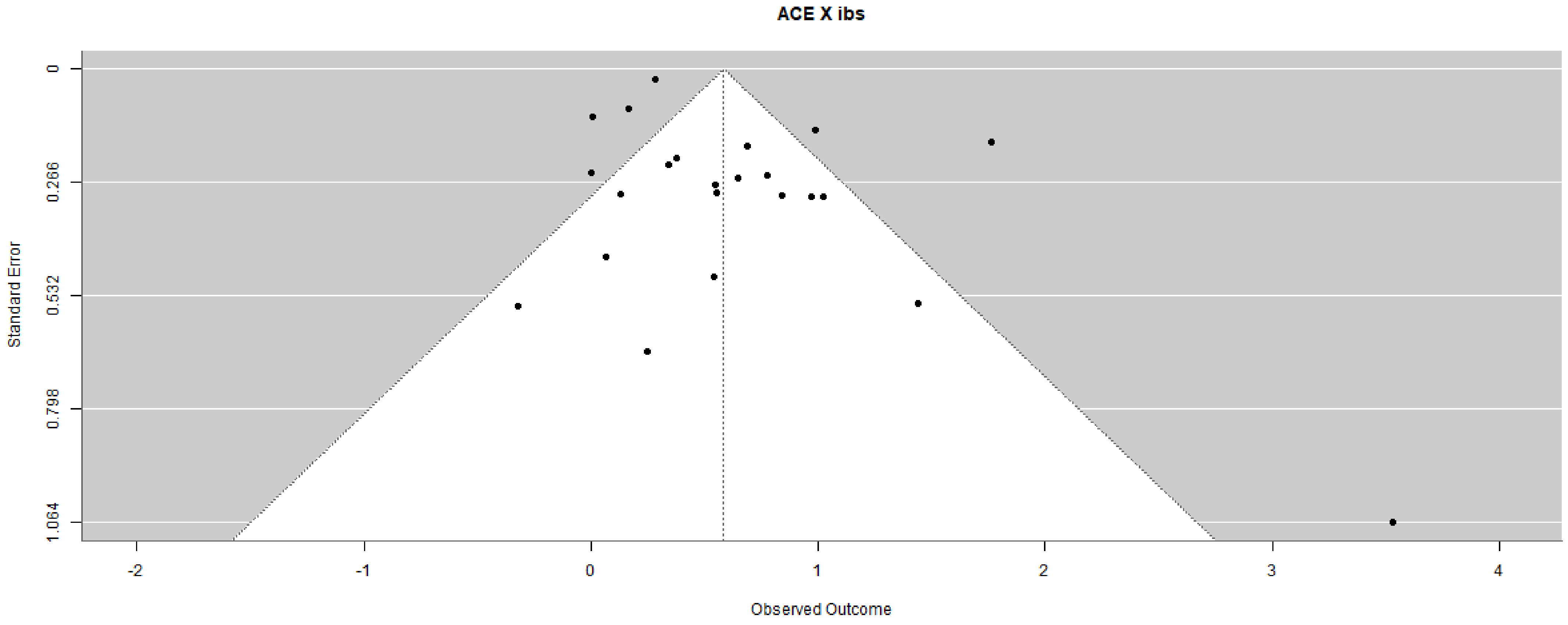

Supplementary Figure 13. Funnel plot for the association between any type of abuse and any type of disease (Class II).

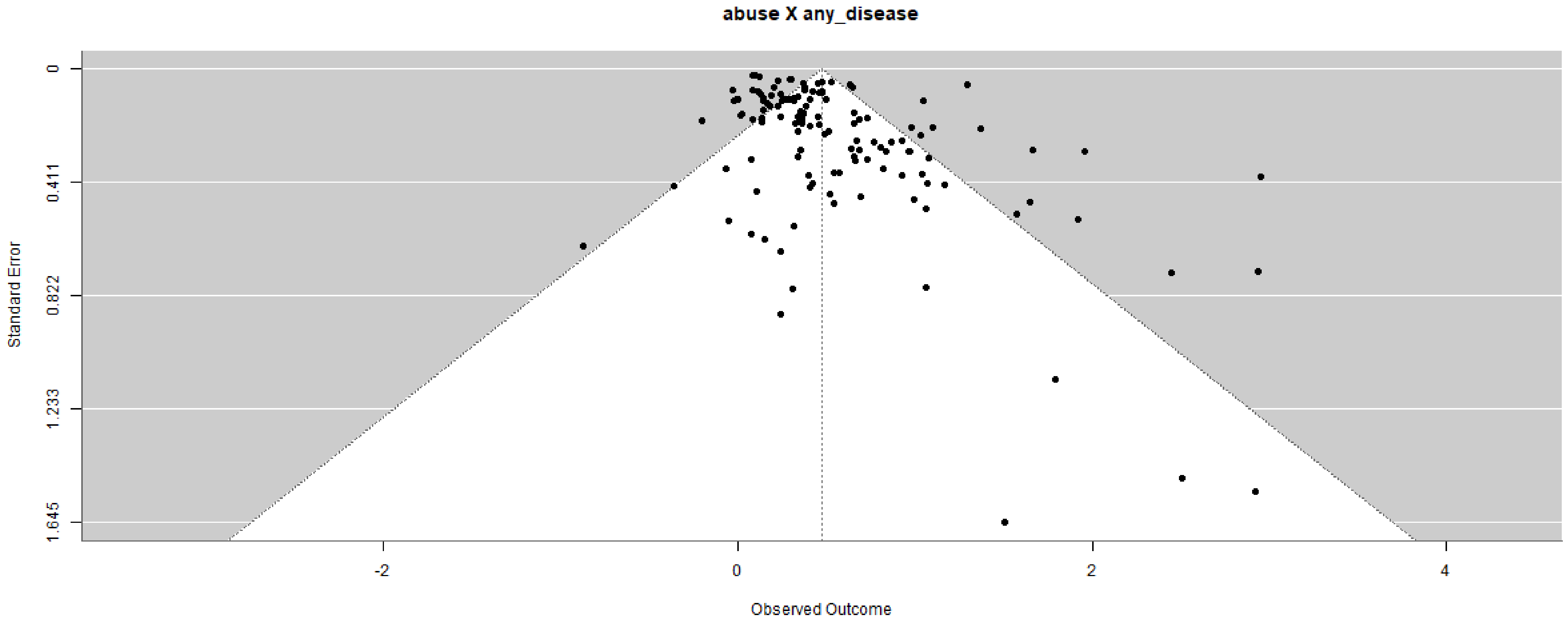

Supplementary Figure 14. Funnel plot for the association between any type of abuse and obesity (Class II).

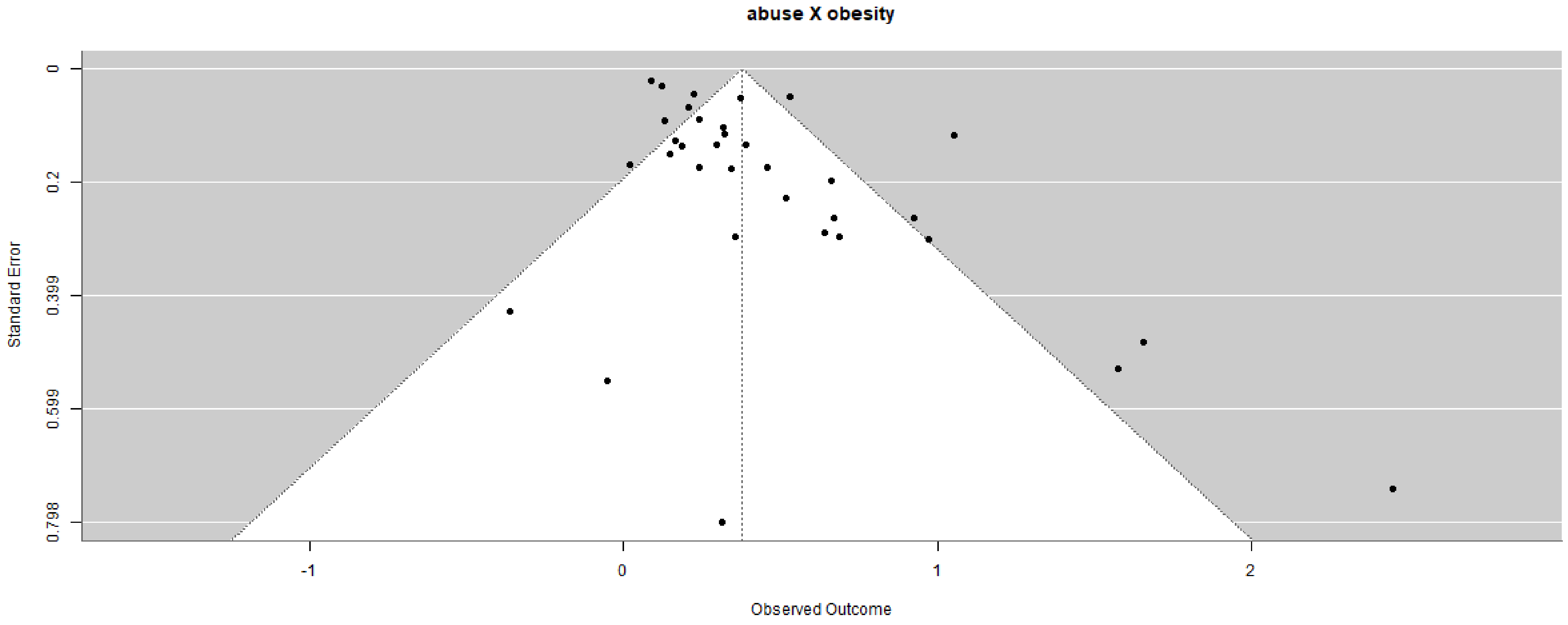

Supplementary Figure 15. Funnel plot for the association between physical abuse and endocrine/nutritional/metabolic diseases (Class II)

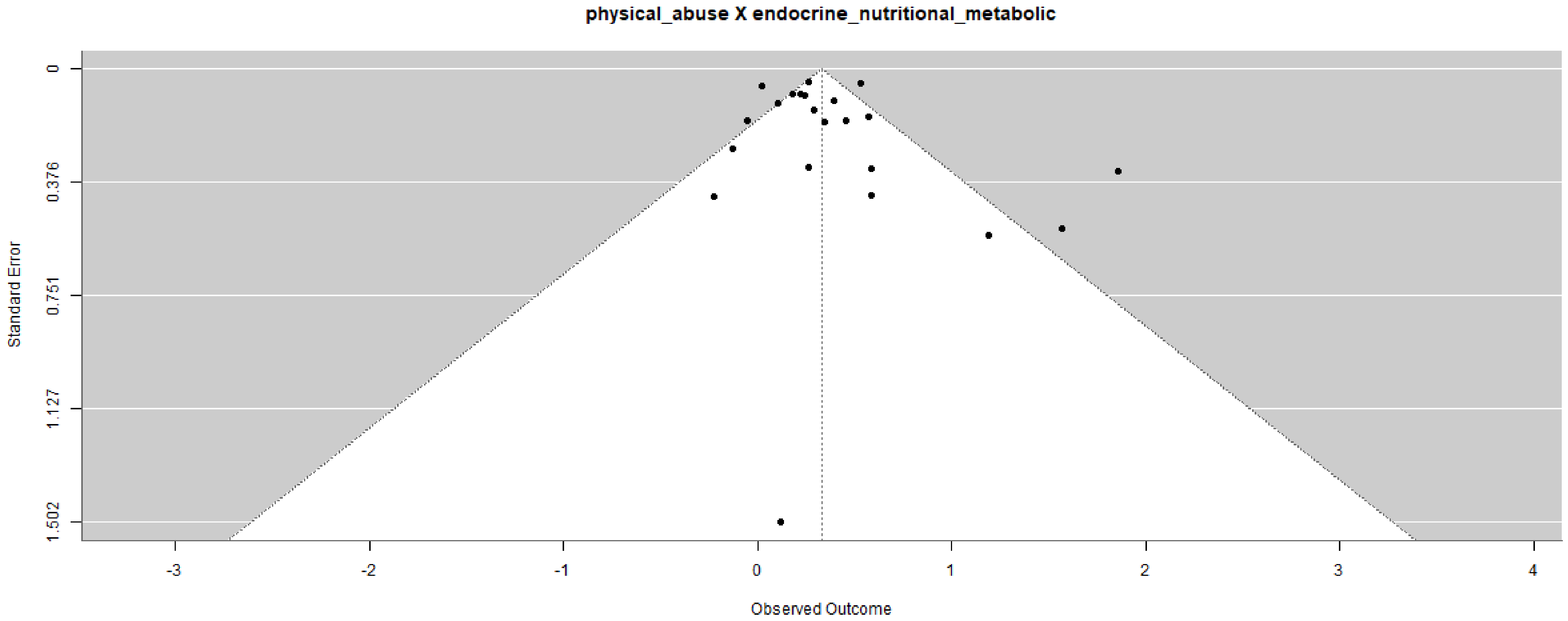

Supplementary Figure 16. Funnel plot for the association between sexual abuse and endocrine/nutritional/metabolic (Class II)

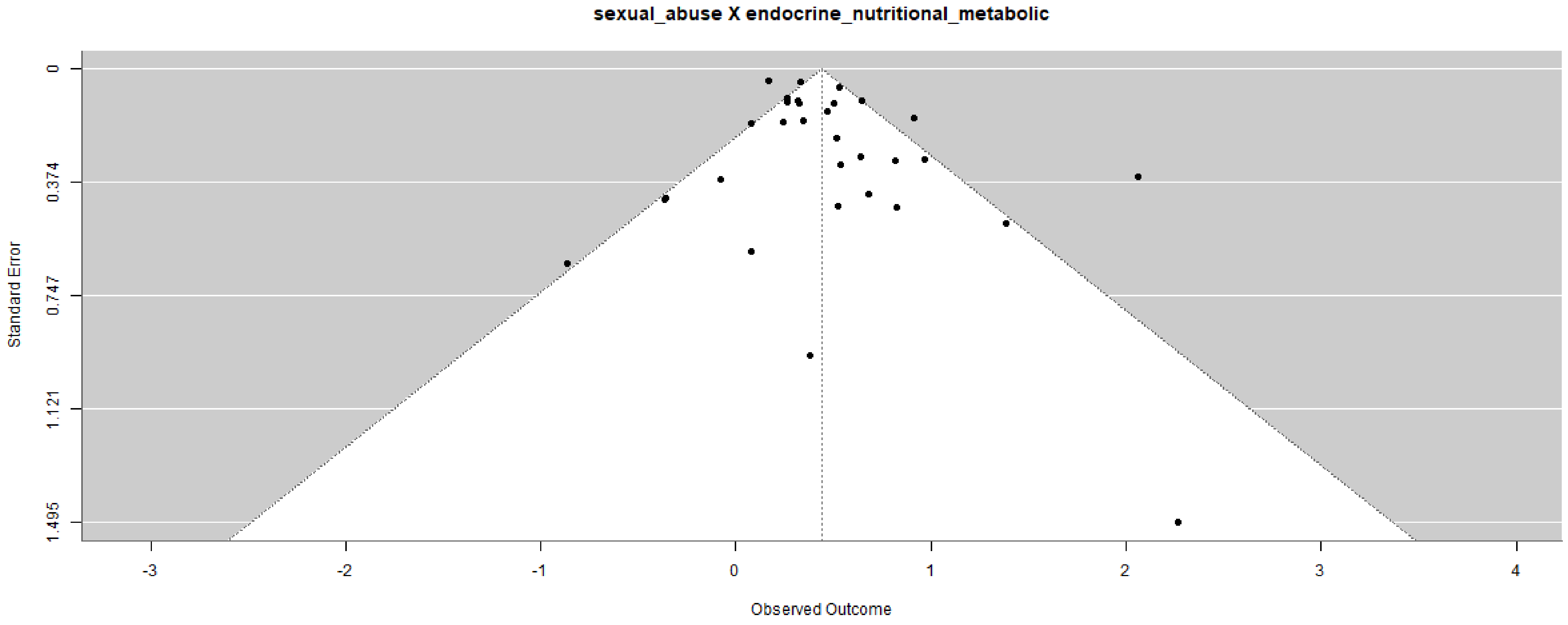

Supplementary Figure 17. Funnel plot for the association between bullying and headache (Class II

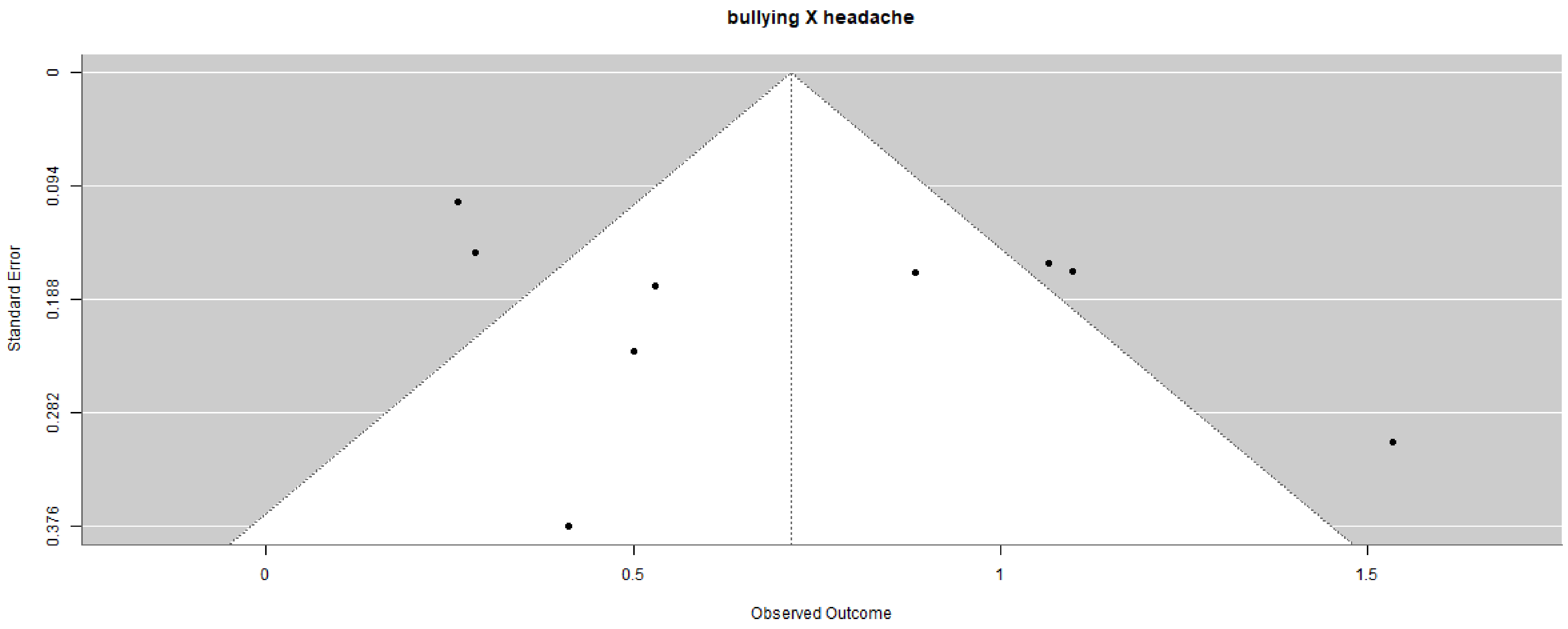

Supplementary Figure 18. Funnel plot for the association between divorce/separation/death and obesity (Class II)

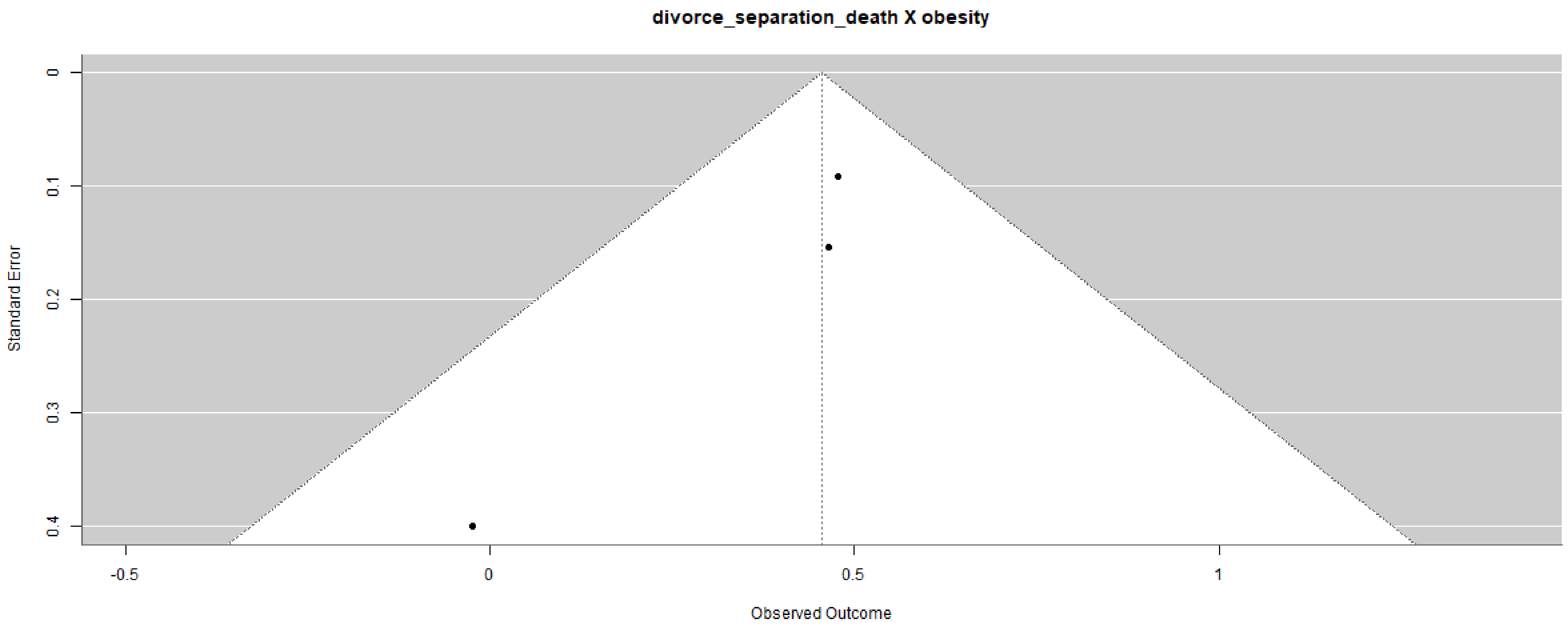

Supplementary Figure 19. Funnel plot for the association between sexual abuse and obesity (Class II)

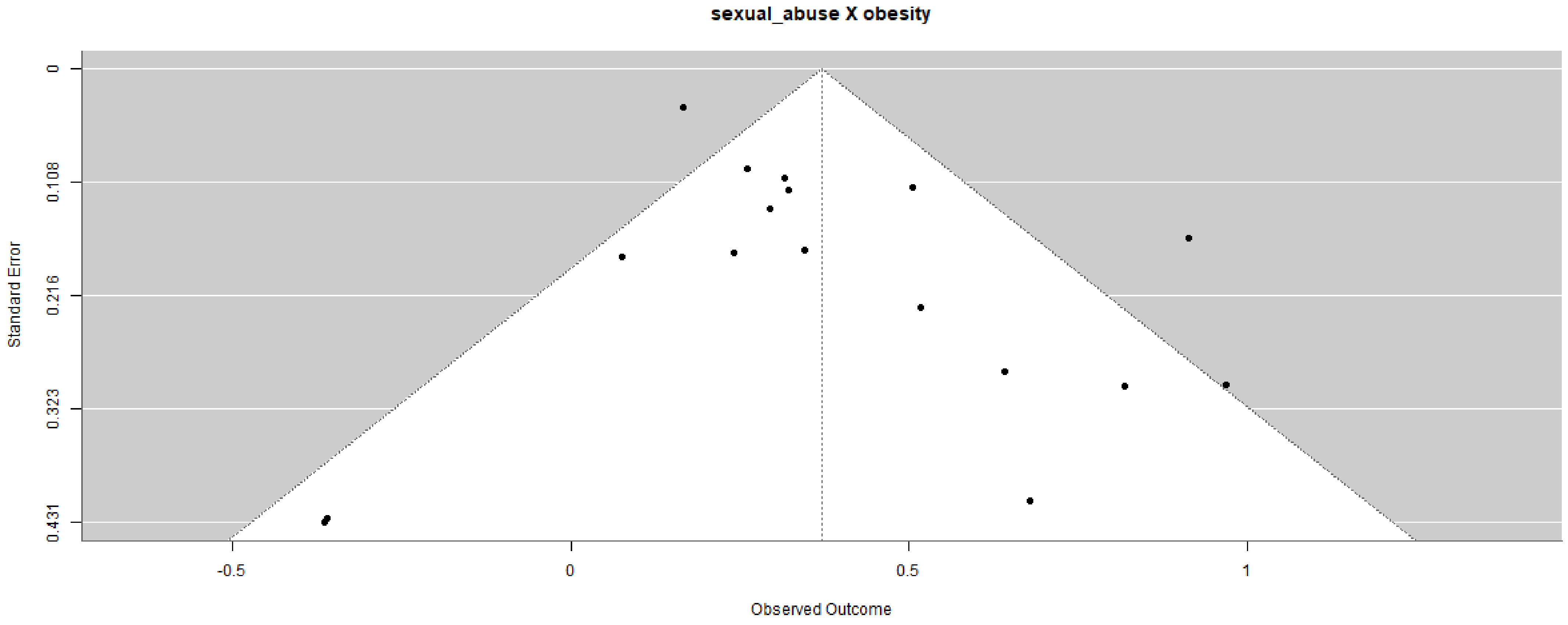

Supplementary Figure 20. Class I association between any type of ACE and physical diseases grouped as endocrine/nutritional/metobolic diseases according to ICD-11 categories, when both trauma and disease are measured with a prospective design

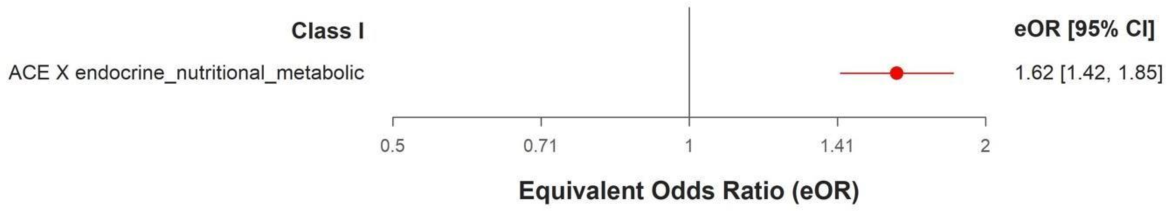

Supplementary Figure 21. Class I association between any type of ACE and physical diseases grouped as circulatory system disorders according to the ICD-11, including only studies which assessed disease prospectively

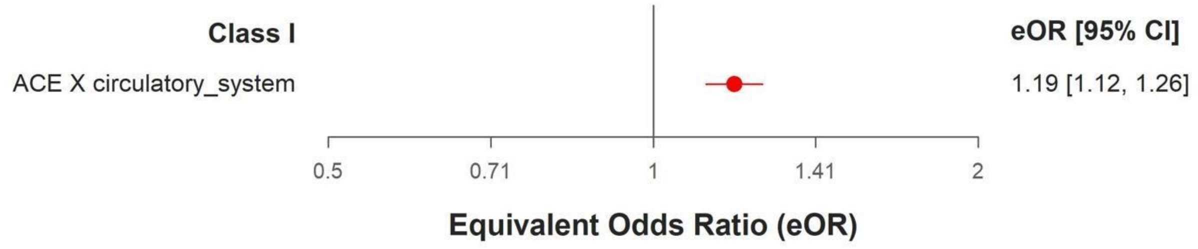

Supplement: Supplementary Information [file mmc1.pdf]
